# Components of Out-of-Pocket Expenditure and Their Relative Contribution to Economic Burden of Diseases in India

**DOI:** 10.1001/jamanetworkopen.2022.10040

**Published:** 2022-05-13

**Authors:** Mayanka Ambade, Rakesh Sarwal, Nachiket Mor, Rockli Kim, S. V. Subramanian

**Affiliations:** 1 International Institute for Population Sciences, Mumbai, India; 2 National Institution for Transforming India Aayog, Government of India, New Delhi, India; 3 Banyan Academy of Leadership in Mental Health, Thiruvidandai, India; 4 Division of Health Policy and Management, College of Health Science, Korea University, Seoul, South Korea; 5 Interdisciplinary Program in Precision Public Health, Department of Public Health Sciences, Graduate School of Korea University, Seoul, South Korea; 6 Harvard Center for Population and Development Studies, Cambridge, Massachusetts; 7 Department of Social and Behavioral Sciences, Harvard T.H. Chan School of Public Health, Boston, Massachusetts

## Abstract

**Importance:**

High out-of-pocket expenditure (OOPE) on health in India may limit achieving universal health coverage. A clear insight on the components of health expenditure may be necessary to make allocative decisions to reduce OOPE, and such details by sociodemographic group and state have not been studied in India.

**Objective:**

To analyze the relative contribution of drugs, diagnostic tests, doctor and surgeon fees, and expenditure on other medical services and nonmedical health-related services, such as transport, lodging, and food, by sociodemographic characteristics of patients, geography, and type of illness.

**Design, Setting, and Participants:**

A population-based cross-sectional health consumption survey conducted by the National Sample Survey Organisation in 2018 was analyzed in this cross-sectional study. Respondents who provided complete information on costs of medicine, doctors, diagnostics tests, other medical costs, and nonmedical costs were selected. Data were analyzed from August through September 2021.

**Main Outcomes and Measures:**

Mean and median share of components (ie, medicine, diagnostic tests, doctor fees, other medical costs, and nonmedical costs) in total health care expenditure and income were calculated. Bivariate survey-weighted mean (with 95% CI) and median (IQR) expenditures were calculated for each component across sociodemographic characteristics. The proportion of total expenditure and income contributed by each cost was calculated for each individual. Mean and median were then used to summarize such proportions at the population level. The association between state net domestic product per capita and component share of each health care service was graphically explored.

**Results:**

Health expenditure details were analyzed for 43 781 individuals for inpatient costs (27 272 [64.3%] women; 26 830 individuals aged 25-64 years [59.9%]) and 8914 individuals for outpatient costs (4176 [48.2%] women; 4901 individuals aged 25-64 years [54.2%]); most individuals were rural residents (24 106 inpatients [67.0]; 4591 outpatients [63.9%]). Medicines accounted for a mean of 29.1% (95% CI, 28.9%-29.2%) of OOPE among inpatients and 60.3% (95% CI, 59.7%-60.9%) of OOPE among outpatients. Doctor consultation charges were a mean of 15.3% (95% CI, 15.1%-15.4%) of OOPE among inpatients and 12.4% (95% CI, 12.1%-12.6%) of OOPE among outpatients. Diagnostic tests accounted for a mean of 12.3% (95% CI, 12.2%-12.4%) of OOPE for inpatient and 9.2% (95% CI, 8.9%-9.5%) of OOPE for outpatient services. Nonmedical costs accounted for a mean of 23.6% (95% CI, 23.3%-23.8%) of OOPE among inpatients and 14.6% (95% CI, 14.1%-15.1%) of OOPE among outpatients. Mean share of OOPE from doctor consultations and diagnostic test charges increased with socioeconomic status. For example, for the lowest vs highest monthly per capita income quintile among inpatients, doctor consultations accounted for 11.5% (95% CI, 11.1%-11.8%) vs 21.2% (95% CI, 20.8%-21.6%), and diagnostic test charges accounted for 10.9% (95% CI, 10.6%-11.1%) vs 14.3% (95% CI, 14.0%-14.5%). The proportion of mean annual health expenditure from mean annual income was $299 of $1918 (15.6%) for inpatient and $391 of $1788 (21.9%) for outpatient services.

**Conclusions and Relevance:**

This study found that nonmedical costs were significant, share of total health care OOPE from doctor consultation and diagnostic test charges increased with socioeconomic status, and annual cost as a proportion of annual income was lower for inpatient than outpatient services.

## Key Points

**Question:**

What are the relative contributions of doctor consultation charges, medicines, diagnostic tests, and nonmedical costs to out-of-pocket expenditure (OOPE) on health care in India?

**Findings:**

This cross-sectional study of 43 781 inpatients and 8914 outpatients found that medicines accounted for 29.1% of inpatient and 60.3% of outpatient OOPE; nonmedical costs, such as travel, lodging, and food, accounted for 23.6% of inpatient and 14.6% of outpatient OOPE; share of OOPE from doctor consultation and diagnostic test charges increased with socioeconomic status; and annual outpatient costs were a greater proportion of annual income of households than annual inpatient costs.

**Meaning:**

These findings suggest that governments may need to focus on nonmedical costs and regulate the drug and diagnostic market.

## Teaser

This cross-sectional study investigates the contributions of doctor consultations, medicines, diagnostic tests, and nonmedical costs to out-of-pocket expenditure on health care in India.

## Introduction

Out of pocket expenditures (OOPEs) may be catastrophic or impoverishing and account for 58.7% of national health expenditure in India.^1,2,3,4,5,6^ Such excessive reliance on OOPE creates a disproportionate burden on low-income individuals, accentuating income inequality and the medical poverty trap.^2^ A 2020 economic survey^3^ estimated that increasing public spending on health to 3% of gross domestic product (GDP) would be associated with a decrease in OOPE to 30% of total health expenditure. However, despite promises by successive governments to increase public health expenditure to 3% of GDP (eg, the Nineismine campaign), India continues to spend 1% of GDP on health care.^3,4^ Nearly 40% to 50% of OOPE is spent on medicines, 10% on diagnostic tests, and 13% on doctor consultations.^5,6,7,8,9,10,11,12^ Between 2004 to 2005 and 2014 to 2015, doctor consultation and diagnostic tests charges increased more than medicine costs.^12^ Nonmedical costs, such as travel and lodging, account for 7% to 17% of OOPE, and this share has sharply increased in the past 2 decades.^7,10^

The current state insurance schemes and tax-funded free or subsidized care interventions are unable to mitigate the burden of OOPE.^13,14,15^ The National Health Policy (2017) aims to correct this and reduce the burden of OOPE (Sustainable Development Goal 3.8.2).^16^ However, researchers have raised concerns about the policy’s implementation, especially fund allocation. It is pertinent to build evidence that justifies the contents of policies. A dissected analysis of health costs may provide a good starting point for doing so.

Currently, evidence from India on components of OOPE is limited. Most of the literature is supplementary and refers to older data sets.^7,8,10,11,12^ Additionally, most estimates do not dissect OOPE into its components by sociodemographic characteristics of patients, geographic location, or diseases. These estimates are essential for informed decision-making on fund allocation and drafting health packages. Therefore, we provided estimates on dissected cost and percentage contribution using the latest round of the health consumption survey (ie, 2018) conducted by National Sample Survey Organisation. To our knowledge, this is the first study to capture the variation in cost dissected by sociodemographic characteristics of patients, their geographic locations, state GDP, chronic morbidities, and type of medical institution. Furthermore, we provide separate estimates for inpatient and outpatient services.

## Methods

This cross-sectional study follows the Strengthening the Reporting of Observational Studies in Epidemiology (STROBE) reporting guideline. The Harvard Longwood Campus Institutional Review Board (IRB) allows researchers to self-determine whether their research meets the requirements of IRB oversight using the IRB Decision Tool. The National Sample Survey data were not collected specifically for this study, and no one on the study team had access to identifiers linked to the data. These activities did not meet the regulatory definition of human participant research, and our study was determined to be exempt from a full institutional review. This study uses secondary information from the National Sample Survey, which acquired informed consent at the time of data collection.^17^ We used a public, deidentified data source^17^ collected by the government of India and had no control in the data-collection process.

This study uses unit records of the Social Consumption: Health survey (seventy-fifth round) conducted by the National Sample Survey Organisation from July 2017 to June 2018. Multistage stratified sampling was performed to collect information about 557 889 individuals from 113 823 households across 14 258 villages and urban blocks. The survey provides details for expenditure on medicines, doctor consultations, diagnostic tests, bed charges for hospitalization, other medical expenses, and nonmedical expenses (eg, transportation, lodging, and food). However, in case a respondent could not recall such details, the survey asked for overall cost termed as a package component. We excluded observations from respondents who reported only package components or had missing sociodemographic information. Details about the sample survey, missing values, and sample selection are available in eFigure 1 and eMethods 1 in the Supplement.

Based on previous evidence,^5,6,7,8,9,10,11,12^ we include sociodemographic and ailment details, such as type of ailment (classification was adopted as given in the survey), age (ie, ages 0-24, 25-64, and ≥65 years), sex (ie, men and women), education level (ie, <secondary, secondary and higher secondary, and ≥graduate), income quintile (ie, lowest, low, middle, high, and highest), caste (ie, general, other backward classes, scheduled castes, and scheduled tribes),^18,19^ residence (ie, rural or urban), and type of facility (ie, public hospital, private hospital, or private clinic). Percentages given for sociodemographic characteristics are weighted and given with an unweighted sample size, as is typically done because weighted percentages represent population distribution; therefore, calculated weighted percentages may differ from percentages as acquired by dividing numerator by denominator (eTable 1 in the Supplement). Caste was self-reported, and classifications for caste were adopted from the questionnaire. The categories other backward castes, scheduled castes, and scheduled tribes were used in the questionnaire as defined by the Constitution of India.^18,19^ The general caste category refers to population not covered under scheduled caste, scheduled tribes, or other backward castes. Castes categories often overlap with socioeconomic categories, and caste is associated with economic and educational opportunities. Individuals belonging to scheduled castes and tribes are often those with low income and education levels. Owing to the tripartite associations among education, income, and caste, all 3 were included in this study as indicators associated with socioeconomic status. More details on ethnic groups (caste) are available in eMethods 2 in the Supplement. Geographical analysis was conducted for major states of India. Here, we grouped northeastern states (ie, Assam, Arunachal Pradesh, Sikkim, Manipur, Meghalaya, Tripura, and Mizoram) and Union territories (Delhi, Chandigarh, Puducherry, Daman and Diu, Dadra and Nagar Haveli, Lakshadweep, and Andaman and Nicobar Islands) into 2 separate groups owing to small sample size. No other geographic region was grouped.

### Statistical Analysis

The basic premise of this study explicates percentage distribution of OOPE into its components. The process involved calculating mean proportions, and 2 approaches were adopted. In the first approach, mean costs were estimated by category and relative proportions were calculated as in Equation 1:
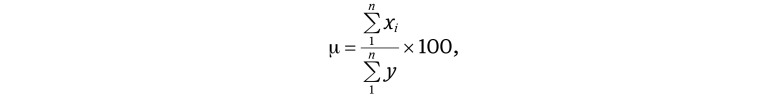
where μ represents the outcome variable, *n* is the number of observations in the population subgroup, *y* represents the total OOPE for the subgroup, and *x_i_* represents the expenditure for a specific component, such as medicine or diagnostic testing, for the subgroup.

Given that the first approach accounts for only aggregate costs, the proportion calculated may overshadow variations at the individual level. Therefore, we calculated percent breakdown for a standard patient, defined as a patient with the mean sociodemographic characteristics of the category in question. Here, first the proportions of each cost (eg, medicine cost) was calculated for individuals and such proportions were then summarized as mean, as in Equation 2:



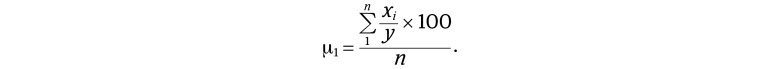



Given that costs are positively skewed, we have calculated median values for robustness. All mean and median values were supplemented with 95% CIs and IQRs, respectively. Moreover, we calculated the relative burden on total income associated with each component.

We also used scatterplots and the Spearman correlation coefficient to investigate the association of state net domestic product with percentage distribution of OOPE components. We used *t* test to calculate 2-sided *P* values with 95% CIs, and statistical significance was set at *P* < .05. Data were analyzed August to September 2021 using Stata statistical software version 14.0 (StataCorp).

## Results

Health expenditure details of 43 781 individuals were analyzed for inpatient costs (27 272 [64.3%] women; 26 830 individuals aged 25-64 years [59.9%]) and 8914 individuals for outpatient costs (4176 [48.2%] women; 4901 individuals aged 25-64 years [54.2%]). Among inpatients, there were 4473 members of a scheduled tribe (6.3%), 7017 members of a scheduled caste (18.5%), 18 437 members of other backward castes (45.5%), and 13 854 members of the general caste (29.6%); among outpatients, there were 626 members of a scheduled tribe (6.7%), 1449 members of a scheduled caste (17.3%), 3524 members of other backward castes (42.5%), and 3315 members of the general caste (33.2%). Most individuals had education below secondary schooling (26 262 inpatients [64.2%]; 6406 outpatients [73.2%]), were rural residents (24 106 inpatients [67.0%]; 4591 outpatients [63.9%]), and used private health care facilities (26 498 inpatients [62.7%]; 5934 outpatients [66.6%]). See eTable 1 in the Supplement for sample distribution for inpatient and outpatient service users.

### Mean Expenditure of Health Care Components

The mean OOPE per capita was estimated at US $299.3 (95% CI, $293.6-$305.1; 21 385 Indian rupees [95% CI, 20 975-21 795 Indian rupees] for inpatient and $390.7 (95% CI, $373.3-$408.2; 27 913 Indian rupees [95% CI, 26 668-29160 Indian rupees]) for outpatient services. Table 1 provides mean annual expenditures (US dollars) on medicines, nonmedical costs, diagnostic tests, doctor consultation fees, and other medical costs for inpatient and outpatient services. Mean annual expenditure for outpatient services was nearly 2-fold that of inpatient services for medicines ($194.6 [95% CI, $187.8-$201.5] vs $84.5 [95% CI, $82.4-$86.6]) and diagnostics ($74.7 [95% CI, $68.0-$81.3 vs $37.9 [95% CI, $37.0-$38.8]). Mean doctor consultation charges were higher for inpatient ($72.5 [95% CI, 70.4-74.6]) than outpatient services ($52.3 [95% CI, $49.2-$55.3). Inpatient and outpatient charges increased with age, education level, and monthly per capita income quintile and were generally higher for men, with a larger increase for inpatient services. Mean expenditure for inpatient services in the private sector was 4-fold higher for medicines ($114.3 [95% CI, $111.1-$117.5] vs $29.7 [95% CI, $28.0-$31.4]) and more than 4-fold higher for diagnostics ($52.2 [95% CI, $51.0-$53.4] vs $11.8 [95% CI, $10.5-$13.0]) compared with the public sector. Mean doctor consultation charges were 12-fold higher in private hospitals ($96.4 [95% CI, $86.8-$105.9]) and 7-fold higher in private clinics ($56.7 [95% CI, $53.9-$59.5]) compared with public hospitals ($8.0 [95% CI, $6.3-$9.7]) for outpatient services and 24-fold higher in private hospitals ($110.0 [95% CI, $106.7-$113.2]) compared with public hospitals ($4.5 [95% CI, $3.6-$5.3]) for inpatient services. Nonmedical costs were similar across patient characteristics for inpatient and outpatient services.

**Table 1.** Mean Component Medical Expenditure by Sociodemographic Characteristic

Characteristic	Cost, mean (95% CI), US $											
	Inpatient services						Outpatient services					
	Individuals, No.	Medicine	Nonmedical costs	Diagnostics	Doctors	Other medical costs	Individuals, No.	Medicine	Nonmedical costs	Diagnostics	Doctors	Other medical costs
Total	43 781	84.5 (82.4-86.6)	32.9 (32.3-33.4)	37.9 (37-38.8)	72.5 (70.4-74.6)	71.3 (69.5-73.2)	8914	194.6 (187.8-201.5)	47.7 (44.3-51.1)	74.7 (68-81.3)	52.3 (49.2-55.3)	21.3 (15.8-26.7)
Age group, y												
0-24	12 782	62.3 (57.9-66.7)	24.7 (24-25.4)	25.9 (24.6-27.2)	46.0 (44.0-48.0)	48.3 (45.4-51.2)	2246	154.1 (140.3-167.8)	40.5 (33.2-47.8)	53.5 (44.2-62.9)	47.3 (43.7-51)	24.6 (5.8-43.4)
25-64	26 830	86.7 (84.4-89)	34.9 (34.1-35.8)	39.1 (38-40.2)	78.5 (75.5-81.5)	75.5 (73.0-78.0)	4901	205.2 (196.3-214.0)	49.0 (44.6-53.4)	88.4 (77.6-99.3)	53.6 (50.3-56.9)	17.4 (14.8-20.1)
≥65	4169	143.0 (133.9-152.1)	46.5 (44.2-48.8)	70.2 (65.6-74.8)	121.5 (112.5-130.5)	120.4 (112.9-127.8)	1767	234.3 (217.4-251.3)	56.6 (49.5-63.6)	67.4 (58.6-76.2)	56.9 (44.2-69.7)	27.9 (21-34.9)
Education level												
<Secondary	26 262	76.3 (74.1-78.5)	31.2 (30.5-31.9)	33.7 (32.5-34.8)	60.4 (58.0-62.7)	62.7 (60.5-64.9)	6406	180.0 (172.0-188.0)	45.8 (42.3-49.3)	53.9 (49.2-58.5)	46.0 (43.4-48.6)	20.7 (13.4-28.1)
Secondary and higher secondary	11 915	89.4 (84-94.8)	33.4 (32.3-34.5)	40.3 (38.7-41.9)	80.9 (76.1-85.7)	75.7 (71.6-79.7)	1693	235.6 (218.6-252.6)	55.9 (45.4-66.4)	156.5 (127.4-185.6)	62.3 (57.4-67.3)	20.7 (16.8-24.6)
≥Graduate	5604	122.6 (116.1-129.1)	42.1 (40.0-44.1)	58.2 (55.2-61.2)	127.2 (119.9-134.6)	113.9 (107.4-120.4)	815	232.3 (212.4-252.2)	45.1 (34.0-56.3)	67.7 (55.5-80.0)	87.2 (60.8-113.6)	27.7 (14.9-40.5)
Monthly per capita income, quintile												
Lowest	8764	63.1 (60.4-65.8)	28.6 (27.4-29.8)	24.9 (23.4-26.5)	50.5 (46.8-54.2)	44.2 (41.6-46.7)	1847	169.1 (154.5-183.8)	40.1 (36.7-43.4)	69.1 (50.9-87.3)	35.6 (32.7-38.6)	11.0 (8.6-13.4)
Low	8919	70.6 (67.3-73.9)	30.8 (29.7-31.9)	29.0 (27.7-30.2)	53.3 (50.2-56.4)	58.3 (54.9-61.7)	1722	181.2 (167.5-195.0)	50.7 (44.4-57.0)	57.5 (44.8-70.1)	41.3 (37.6-45.0)	27.8 (2.6-53.0)
Middle	9996	79 (75.4-82.5)	32 (31.0-33.1)	35.4 (33.9-36.9)	65.7 (61.6-69.7)	64.8 (62.0-67.6)	1887	188.7 (176.5-200.9)	54.4 (44.5-64.4)	58.4 (51.5-65.4)	47.0 (42.7-51.3)	22.2 (16.7-27.7)
High	7475	107.3 (101.4-113.3)	39.2 (37.5-41.0)	47.1 (44.3-49.9)	85.6 (81.3-89.9)	90.4 (85.4-95.5)	1676	223.8 (208.1-239.5)	41.1 (36.5-45.7)	94.9 (83.0-106.8)	76.5 (65.3-87.6)	29.4 (23.7-35.1)
Highest	8627	124.8 (116.6-132.9)	37.7 (36.2-39.2)	66.6 (63.6-69.7)	133.9 (125.9-141.9)	125.1 (117.9-132.2)	1782	236.8 (216.3-257.4)	56.4 (44.3-68.4)	107.3 (87.8-126.7)	79.1 (68.2-89.9)	21.5 (14.8-28.2)
Sex												
Men	16 509	118.7 (113.7-123.7)	42.8 (41.5-44.0)	51.6 (49.7-53.4)	97.5 (93.2-101.7)	99.5 (95.5-103.6)	4738	183.9 (175.1-192.6)	46.2 (40.7-51.8)	74.1 (62.6-85.5)	51.7 (47.9-55.4)	24.3 (14.2-34.3)
Women	27 272	65.5 (63.9-67.1)	27.4 (26.9-27.9)	30.3 (29.4-31.2)	58.7 (56.5-60.9)	55.7 (53.9-57.5)	4176	206.2 (195.5-216.9)	49.3 (45.7-52.9)	75.3 (69.3-81.3)	52.9 (47.9-57.9)	18.0 (14.9-21.2)
Caste												
General^a^	13 854	98.0 (94.2-101.8)	34.7 (33.6-35.8)	47.8 (45.7-49.9)	89.2 (84.7-93.8)	88.1 (83.9-92.2)	6051	202.8 (190.0-215.6)	47.0 (40.8-53.2)	86.0 (75.1-97.0)	64.7 (57.7-71.8)	17.1 (13.2-21.1)
Other backward caste	18 437	83.3 (79.9-86.8)	32.4 (31.6-33.1)	35.8 (34.7-36.9)	72.1 (69.1-75.2)	68.6 (66.0-71.2)	4941	200.6 (189.9-211.3)	49.7 (45.5-53.9)	61.5 (54.0-69.0)	48.3 (44.5-52.2)	23.9 (11.1-36.6)
Scheduled caste	7017	75.1 (70.7-79.6)	31.5 (30.0-33.1)	33.7 (31.7-35.6)	58.4 (53.9-63.0)	59.6 (55.8-63.4)	3853	184.3 (170.4-198.1)	44.1 (33.1-55.0)	100.2 (74.0-126.4)	41.9 (38.2-45.6)	23.2 (18.9-27.6)
Scheduled tribe	4473	56.5 (50.0-63.0)	32.1 (30.2-34.0)	19.6 (17.9-21.2)	38.7 (35.2-42.2)	47.1 (41.7-52.5)	2515.09	144.0 (124.1-163.9)	48.1 (41.4-54.8)	36.1 (27.4-44.7)	42.3 (34.8-49.9)	20.1 (13.5-26.6)
Residence												
Rural	24 106	76.6 (74.5-78.8)	33.8 (33.1-34.6)	32.6 (31.4-33.7)	61.7 (59.3-64.0)	60.9 (58.9-62.8)	4591	184.2 (174.8-193.6)	48.7 (44.4-52.9)	63.3 (54.1-72.5)	40.4 (38.1-42.8)	18.0 (9.0-27.0)
Urban	19 675	100.4 (96.1-104.7)	30.9 (30.1-31.8)	48.7 (47.3-50.2)	94.7 (90.7-98.7)	92.7 (88.9-96.4)	4323	213.3 (203.1-223.4)	46.0 (40.4-51.5)	94.9 (85.2-104.5)	73.3 (66.8-79.9)	27.1 (22.8-31.3)
Facility^b^												
Public hospital	16 180	29.7 (28-31.4)	20.9 (20.3-21.5)	11.8 (10.5-13)	4.5 (3.6-5.3)	13.2 (11.9-14.5)	2154	134.1 (120.5-147.8)	46.6 (42.5-50.6)	22.4 (17.6-27.2)	8.0 (6.3-9.7)	15.7 (12.4-19.0)
Private hospital	26 498	114.3 (111.1-117.5)	39.2 (38.4-40)	52.2 (51.0-53.4)	110.0 (106.7-113.2)	103.3 (100.5-106.2)	2811	314.8 (297.4-332.2)	79.4 (70.8-88.0)	148.9 (130.2-167.7)	96.4 (86.8-105.9)	47.1 (28.5-65.7)
Private clinic	NA	NA	NA	NA	NA	NA	3123	178.8 (170.7-186.9)	37.1 (31.8-42.5)	65.8 (56.8-74.4)	56.7 (53.9-59.5)	12.4 (9.8-15.0)

Abbreviation: NA, not applicable.

^a^ Other backward castes, scheduled castes, and scheduled tribes were identified as defined by the government of India. General caste category refers to population not covered under scheduled caste, scheduled tribes, or other backward castes.

^b^ Values for nongovernmental organization–run hospitals are not provided here for inpatient services or for nongovernental organizations or traditional healers for outpatient services.

### Mean Proportion of Total Health Expenditure by Health Care Component Cost

The Figure and Table 2 present overall mean percentage share of OOPE for all components. Medicines continued to account for the largest mean percentage of OOPE (29.1% [95% CI, 28.9%-29.2%] for inpatient services and 60.3% [95% CI, 59.7%-60.9%] for outpatient services), followed by nonmedical costs (23.6% [95% CI, 23.3%-23.8%] for inpatient and 14.6% [95% CI, 14.1%-15.1%] for outpatient services), other medical costs (19.5% [95% CI, 19.4-19.7%] for inpatient and 3.3% [95% CI, 3.1%-3.6%] for outpatient services), doctor consultation charges (15.3% [95% CI, 15.1%-15.4%] for inpatient and 12.4% [95% CI, 12.1%-12.6%] for outpatient services), and diagnostic tests (12.3% [95% CI, 12.2%-12.4%] for inpatient and 9.2% [95%, 8.9%-9.5%] for outpatient services).

**Figure.**
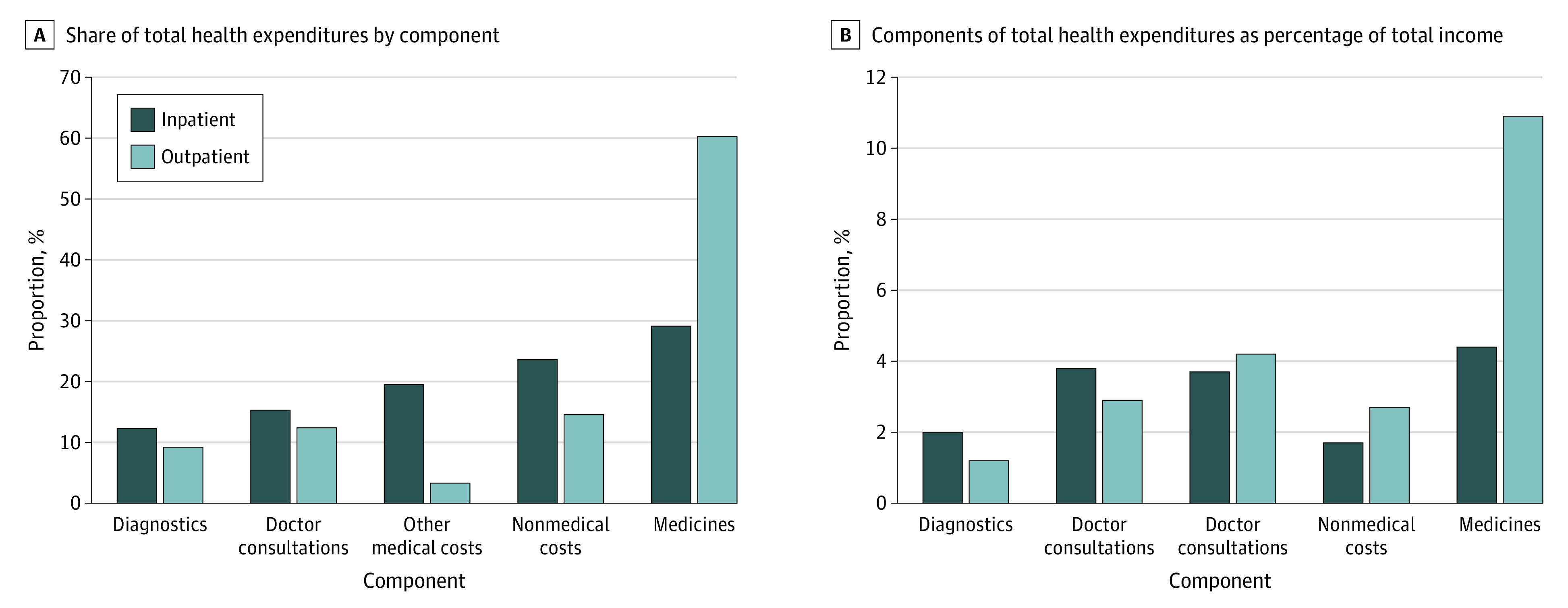
Component Costs as Percentage of Total Health Expenditures and Total Income

**Table 2.** Proportion of Total Health Expenditure From Service Components by Sociodemographic Characteristic

Characteristic	Proportion share, mean (95% CI), %													
	Inpatient services							Outpatient services						
	Individuals, No.	Total	Medicine	Nonmedical costs	Diagnostics	Doctors	Other medical costs	Individuals, No.	Total	Medicine	Nonmedical cost	Diagnostics	Doctors	Other medical costs
Total	43781	100	29.1 (28.9-29.2)	23.6 (23.3-23.8)	12.3 (12.2-12.4)	15.3 (15.1-15.4)	19.5 (19.4-19.7)	8914	100	60.3 (59.7-60.9)	14.6 (14.1-15.1)	9.2 (8.9-9.5)	12.4 (12.1-12.6)	3.3 (3.1-3.6)
Age group, y														
0-24	12782	100	28.6 (28.3-28.9)	26.5 (26.0-27.0)	11.6 (11.4-11.8)	13.9 (13.6-14.2)	19.2 (18.9-19.4)	2246	100	58.0 (56.9-59.2)	15.7 (14.8-16.7)	8.2 (7.6-8.7)	14.5 (13.9-15.1)	3.4 (2.8-3.9)
25-64	26830	100	29.2 (29.0-29.5)	22.9 (22.6-23.2)	12.4 (12.3-12.6)	15.8 (15.6-16.0)	19.4 (19.2-19.6)	4901	100	61.2 (60.4-62.1)	13.9 (13.3-14.5)	9.9 (9.5-10.4)	11.7 (11.3-12.1)	2.9 (2.6-3.3)
≥65	4169	100	29.5 (28.9-30.0)	18.2 (17.6-18.8)	13.9 (13.6-14.3)	16.6 (16.1-17.1)	21.6 (21.1-22.0)	1767	100	61.5 (60.0-63.0)	14.7 (13.7-15.8)	8.5 (7.9-9.2)	10.4 (9.8-11.1)	4.6 (3.9-5.3)
Education level														
<Secondary	26262	100	29.9 (29.6-30.1)	25.3 (25.0-25.7)	12 (11.9-12.2)	13.6 (13.4-13.8)	19 (18.8-19.1)	6406	100	60.8 (60.1-61.6)	15.5 (14.9-16.0)	8.3 (8.0-8.7)	11.8 (11.5-12.2)	3.3 (3.0-3.6)
Secondary and higher secondary	11915	100	28.0 (27.7-28.4)	21.9 (21.5-22.3)	12.8 (12.6-13.0)	17.0 (16.7-17.3)	20.0 (19.7-20.2)	1693	100	58.3 (56.9-59.7)	13.2 (12.2-14.3)	12.5 (11.6-13.3)	12.8 (12.1-13.4)	3.0 (2.4-3.5)
≥Graduate	5604	100	26.6 (26.2-27.1)	16.6 (16.0-17.2)	12.8 (12.5-13.1)	21.6 (21.1-22.1)	22.1 (21.7-22.4)	815	100	60.1 (58.0-62.3)	9.5 (8.4-10.7)	9 (8.0-9.9)	16.4 (15.2-17.6)	4.7 (3.7-5.7)
Monthly per capita income, quintiles														
Lowest	8764	100	31.7 (31.3-32.1)	29.9 (29.3-30.5)	10.9 (10.6-11.1)	11.5 (11.1-11.8)	15.7 (15.4-16.1)	1847	100	62.7 (61.4-64.1)	15.5 (14.5-16.5)	8.1 (7.4-8.7)	10.4 (9.8-11.0)	3 (2.5-3.6)
Low	8919	100	29.3 (28.9-29.7)	26.4 (25.9-27.0)	11.9 (11.7-12.2)	13.2 (12.9-13.5)	18.9 (18.6-19.2)	1722	100	61.7 (60.3-63.1)	14.7 (13.7-15.6)	8.4 (7.7-9.1)	11.2 (10.6-11.8)	3.7 (3.1-4.3)
Middle	9996	100	28.7 (28.3-29.1)	23.0 (22.5-23.5)	12.3 (12.1-12.6)	15.3 (15.0-15.7)	20.4 (20.1-20.7)	1887	100	57.5 (56.1-58.8)	17.0 (15.9-18.2)	8.4 (7.8-9.0)	12.9 (12.3-13.5)	4.0 (3.4-4.6)
High	7475	100	27.6 (27.2-27.9)	19.1 (18.6-19.7)	13.2 (13.0-13.5)	18.4 (18.1-18.8)	21.4 (21.1-21.7)	1676	100	58.7 (57.2-60.2)	12.5 (11.5-13.5)	11.8 (11.0-12.6)	13.7 (13.0-14.5)	3.0 (2.5-3.5)
Highest	8627	100	26.5 (26.1-26.8)	14.3 (13.9-14.7)	14.3 (14.0-14.5)	21.2 (20.8-21.6)	23.6 (23.3-23.9)	1782	100	59.1 (57.7-60.5)	12 (11.1-13.0)	10.5 (9.7-11.2)	15.3 (14.6-16.1)	2.8 (2.3-3.3)
Sex														
Men	16509	100	30.2 (29.9-30.4)	19.0 (18.6-19.3)	13.0 (12.9-13.2)	16.3 (16.0-16.5)	21.3 (21.1-21.6)	4738	100	60.6 (59.7-61.4)	14.1 (13.5-14.7)	8.7 (8.3-9.1)	13.0 (12.6-13.5)	3.4 (3.1-3.8)
Women	27272	100	28.5 (28.2-28.7)	26.1 (25.8-26.4)	11.9 (11.8-12.1)	14.7 (14.5-14.9)	18.5 (18.4-18.7)	4176	100	60.0 (59.1-60.9)	15.1 (14.4-15.8)	9.7 (9.3-10.2)	11.6 (11.2-12.0)	3.2 (2.9-3.6)
Caste^a^														
General	13854	100	28.6 (28.3-28.9)	20.0 (19.6-20.4)	13.3 (13.1-13.5)	17.0 (16.8-17.3)	20.8 (20.5-21.1)	3315	100	60.7 (59.7-61.8)	12.5 (11.8-13.1)	10.2 (9.6-10.7)	13.6 (13.1-14.1)	2.8 (2.4-3.2)
Other backward castes	18437	100	28.7 (28.4-29.0)	22.9 (22.5-23.2)	12.1 (12.0-12.3)	16.3 (16.0-16.5)	19.8 (19.6-20.0)	3524	100	61.5 (60.5-62.4)	14.3 (13.6-15.0)	8.6 (8.1-9.0)	12.4 (11.9-12.8)	3.0 (2.6-3.4)
Scheduled caste	7017	100	30.9 (30.4-31.4)	26.3 (25.7-26.9)	12.1 (11.8-12.4)	12.1 (11.7-12.4)	18.4 (18.0-18.8)	1449	100	59.6 (58-61.2)	15.4 (14.2-16.6)	9.6 (8.8-10.4)	10.7 (10.0-11.4)	4.5 (3.8-5.2)
Scheduled tribe	4473	100	28.4 (27.8-29)	37.2 (36.3-38.1)	9.8 (9.4-10.1)	9.3 (8.8-9.7)	15.1 (14.6-15.6)	626	100	52.8 (50.3-55.4)	24.5 (22.1-27.0)	7.1 (6.1-8.0)	10.4 (9.4-11.5)	4.9 (3.7-6.1)
Place of residence														
Rural	24106	100	29.6 (29.3-29.8)	26.2 (25.9-26.5)	11.8 (11.6-11.9)	13.8 (13.6-14.0)	18.4 (18.2-18.6)	4591	100	61.8 (61-62.7)	15.5 (14.9-16.1)	8.4 (8.0-8.8)	10.9 (10.5-11.2)	3.2 (2.8-3.5)
Urban	19675	100	28.0 (27.8-28.3)	18.2 (17.9-18.5)	13.4 (13.2-13.5)	18.3 (18.1-18.6)	21.8 (21.6-22.0)	4323	100	57.6 (56.7-58.5)	12.9 (12.3-13.6)	10.6 (10.1-11.1)	15.0 (14.5-15.5)	3.6 (3.2-4.0)
Type of facility^b^														
Public hospital	16180	100	30.7 (30.3-31.1)	45.5 (45.0-45.9)	10.4 (10.1-10.6)	2.0 (1.8-2.1)	11.2 (11.0-11.5)	2154	100	55.1 (53.5-56.7)	34.3 (32.8-35.9)	4.5 (4.0-5.0)	1.7 (1.4-2.0)	4.1 (3.5-4.7)
Private hospital	26498	100	28.1 (28-28.3)	11.6 (11.5-11.7)	13.4 (13.3-13.5)	22.5 (22.3-22.7)	24.1 (23.9-24.3)	2811	100	55.0 (54.1-55.9)	11.3 (10.8-11.7)	13.9 (13.3-14.5)	16.0 (15.5-16.5)	3.7 (3.2-4.1)
Private clinic	NA	NA	NA	NA	NA	NA	NA	3123	100	60.6 (59.6-61.5)	8.9 (8.5-9.3)	10.0 (9.5-10.5)	17.3 (16.8-17.8)	3.1 (2.6-3.5)

Abbreviation: NA, not applicable.

^a^ Other backward castes, scheduled castes, and scheduled tribes were identified as defined by the Constitution government of India. General caste category refers to population not covered under scheduled caste, scheduled tribes, or other backward castes.

^b^ Values for nongovernmental organization–run hospitals are not provided here for inpatient services or for nongovernmental organizations or traditional healers for outpatient services.

Table 2 provides the percentage breakup by sociodemographic category. The mean share of OOPE from doctor consultation charges was higher among residents who were educated (eg, ≥graduate vs <secondary education: 21.6% [95% CI, 21.1%-22.1%] vs 13.6 (95% CI, 13.4%-13.8%) for inpatient services and 16.4% [95% CI, 15.2%-17.6%] vs 11.8 [95% CI, 11.5%-12.2%] for outpatient services), high income, (eg, highest vs lowest monthly per capita income quintile: 21.2% [95% CI, 20.8%-21.6%] vs 11.5% [95% CI, 11.1%-11.8% for inpatient services and 15.3% [95% CI, 14.6%-16.1%] vs 10.4% [95% CI, 9.8%-11.0%] for outpatient services), and urban vs rural residents (18.3% [95% CI, 18.1%-18.6%] vs 13.8% [95% CI, 13.6%-14.0%] for inpatient services and 15.0% [95% CI, 14.5%-15.5%] vs 10.9% [95% CI, 10.9%-11.5%] for outpatient services), with greater increases for outpatient services. Mean doctor consultation charges contributed a trivial amount to OOPE in public facilities (2.0% [95% CI, 1.8%-2.1%] for inpatient and 1.7% [95% CI, 1.4%-2.0%] for outpatient services). The mean relative share of OOPE from diagnostic tests was higher for individuals aged 65 years and older (eg, vs individuals aged 0-24 years: 13.9% [95% CI, 13.6%-14.3%] vs 11.6% [95% CI, 11.4%-11.8%]) for inpatient services and ages 25 to 64 years (eg, vs ages ≥65 years: 9.9% [95% CI, 9.5%-10.4%] vs 8.5% [95% CI, 7.9%-9.2%] for outpatient services), higher income individuals (eg, highest vs lowest monthly per capita income: 14.3% [95% CI, 14.0%-14.5%] vs 10.9% [95% CI, 10.6%-11.1%] for inpatient services and 10.5% [95% CI, 9.7%-11.2%] vs 8.1% [95% CI, 7.4%-8.7%] for outpatient services), and those using private facilities (eg, private vs public hospitals for inpatient services: 13.4% [95% CI, 13.3%-13.5%] vs 10.4% [95% CI, 10.1%-10.6%] and private clinics vs public hospitals for outpatient services: 10.0% [95% CI, 9.5%-10.5%] vs 4.5% [4.0%-5.0%]. It was lower among scheduled tribes (eg, vs general caste: 9.8% [95% CI, 9.4%-10.1%] vs 13.3% [95% CI, 13.1%-13.5%] for inpatient services and 7.1% [95% CI, 6.1%-8.0%] vs 10.2% [9.6%-10.7%] for outpatient services), and rural residents vs urban residents (11.8% [95% CI, 11.6%-11.9%] vs 13.4% [95% CI, 13.2%-13.5%] for inpatient services and 8.4% [95% CI, 8.0%-8.8%] vs 10.6% [95% CI, 10.1-11.1%] for outpatient services). Mean shares of OOPE from other medical costs remained comparable across most categories. Mean contribution of nonmedical expenditures decreased with age, education, and income for inpatient and outpatient services. Additionally, it was higher for public facilities (eg, vs private hospitals for outpatient services: 34.3% [95% CI, 32.8%-35.9%] vs 11.3% [95% CI, 10.8%-11.7%), scheduled tribes (eg, vs general caste: 37.2 [95% CI, 36.3%-38.1%] vs 20.0% [95% CI, 19.6%-20.4%] for inpatient and 24.5% [95% CI, 22.1%-27.0%] vs 12.5% [95% CI, 11.8%-13.1%] for outpatient services ), rural vs urban residents (26.2% [95% CI, 25.9%-26.5%] vs 18.2% [17.9%-18.5%] for inpatient and 15.5% [95% CI, 14.9%-16.1% vs 12.9% [95% CI, 12.3%-13.6% for outpatient services).

The mean relative contribution of each component to OOPE as calculated by method 1 confirmed the trends as presented in Table 2 to some extent (eTable 2 in the Supplement). For inpatient services the contribution to the $299.3 (21 385 Indian rupees) mean total OOPE was highest for medicines ($84.5 [6037 Indian rupees; 28.2%]), followed by doctor consultations ($72.5 [5185 Indian rupees; 24.2%]), other medical costs ($71.3 [5099 Indian rupees; 23.8%]), diagnostics ($37.9 [2711 Indian rupees; 12.7%]), and nonmedical fees ($32.9 [2352 Indian rupees; 11.0%]). For outpatient services, the contribution of components to the $390.7 (27 914 Indian rupees) mean total OOPE was highest for medicines ($194.6 [13 907 Indian rupees; 49.8%]), followed by diagnostics ($74.7 [5337 Indian rupees; 19.1%]), doctor consultation charges ($52.3 [3737 Indian rupees; 13.4%]), nonmedical costs ($47.7 [3411 Indian rupees; 12.2%]), and other medical charges ($21.3 [1522 Indian rupees; 5.4%]).

For robustness, we calculated the mean proportion for each cost for inpatient and outpatient services by including all missing values (eTable 3 in the Supplement). The mean percentage contribution to OOPE of medicines, diagnostic tests, and doctor fees was similar for inpatient and outpatient services. However, nonmedical costs accounted for a mean 35.7% (95% CI, 35.4%-35.9%) of OOPE for inpatient and 17.6% (95% CI, 17.3%-17.8%) for outpatient services.

### Median Proportion of OOPE From Health Care Component Costs

The median (IQR) percentage of OOPE (eTable 4 in the Supplement) values show that 50% of the population had no expenditures on diagnostic tests (0% [0%-16.1%]) and other medical needs (0%) for outpatient services. The median (IQR) percentage of OOPE for nonmedical costs was 7.4% (0%-18.0%) for outpatient and 13.7% (7.3%-28.3%) for inpatient services. However, 50% of the population spent more than 60% of OOPE on medicines alone (outpatients: 60.4% (40.6%-86.9%). The highest median (IQR) percentage among outpatient services for nonmedical fees was found among individuals using public hospitals for (eg, vs private hospitals: 18.5%; [3.8%-54.6%] vs 8.4% [2.9%-16.0%]), scheduled tribes (eg, vs general caste: 13.3% [0%-31.8%] vs 6.0 [0%-16.6%]), and individuals who were low income (eg, low vs highest per capita monthly income: 9.0% [0%-19.3%] vs 5.3% (0%-13.4%). For inpatient services in the public setting, median (IQR) percentage of OOPE was as high as 37% (37.8% [20.3%-66.6%] for nonmedical costs).

### Health Care Component Costs as a Proportion of Income

The relative contribution of each cost and total OOPE as a proportion of household total income (represented by consumption expenditure in National Sample Surveys) is provided in eTable 5 in the Supplement. The proportion of mean annual income accounted for by mean annual health expenditure was $299 of $1918 (21 385 of 13 7012 Indian rupees [15.6%]) for inpatient and $391 of $1788 (27 914 of 127 738 Indian rupees [21.9%]) for outpatient services. Medicines contributed the highest proportion of annual income for inpatient ($84.5 [6037 Indian rupees; 4.4%]) and outpatient services ($195 [13 907 Indian rupees; 10.9%]). For inpatient services, doctor consultation charges accounted for $72.5 (5185 Indian rupees; 3.8%) of income, followed by other medical costs ($71.3 [5099 Indian rupees; 3.7%]), diagnostic tests ($37.9 [2711 Indian rupees; 2.0%]), and nonmedical costs ($32.9 [2352 Indian rupees; 1.7%]). For outpatient services, diagnostic charges accounted for $74.7 (5337 Indian rupees; 4.2%) of income, followed by doctor charges ($52.3 [3737 Indian rupees; 2.9%]), nonmedical costs ($47.7 [3411 Indian rupees; 2.7%]), and other medical costs ($21.3 [1552 Indian rupees; 1.2%]). The trend for inpatient and outpatient services by age, education, sex, income, and caste was similar to results in Table 2 and eTable 2 in the Supplement.

### Variation by State and Ailment Wise Variation

Table 3 provides the statewise percentage distribution of health expenditure into components and state-level health expenditure (in millions of $) as a percentage of total state GDP (in millions of $) in 2017 to 2018 from National Health Accounts.^1^ States that assigned less than 1% of their GDP to health, such as Telangana ($816.5 of $97 906.5 [0.8%]) had lower contributions from medicines and nonmedical costs and higher contributions from doctor consultation charges and diagnostic tests than states that assigned more than 1% of GDP to health, such as Bihar ($852.9 of $63016.2 [1.4%]). For example, the proportion of OOPE from medicines among inpatients in Telangana was lower than in Bihar (23.6% [95% CI, 22.8%-24.3%] vs 33.3% [95% CI, 32.5%-34.1%]), whereas the contribution of doctor consultation to OOPE was higher in Telangana than in Bihar (24.4% [95% CI, 23.5%-25.3%] vs 12.4% [95% CI, 11.7%-13.1%]). Furthermore, the share of OOPE from nonmedical costs ranged from 15.3% (95 % CI, 14.8%-15.8%) in Karnataka (health expenditure = $1247.2 of $176 485.3 state GDP [0.7%]) to 43.3% (95% CI, 41.3%-45.2%) in Chhattisgarh (health expenditure = $551.7 of $36 945.2 state GDP [1.5%]) for inpatient services and from 7.9% (95% CI, 6.7%-9.0%) in West Bengal (health expenditure = $1357.5 of $129 946.1 state GDP [1.0%]) to 35.5% (95% CI, 29.1%-41.9%) in Uttarakhand ($232.4 of $28 968.7 [0.8%]) for outpatient services.

**Table 3.** Proportion of Total Health Expenditure From Service Components by State

State	Gross state domestic product, US $million^a^	Gross health expenditure, US $million (%)^a^	Mean proportion, % (95% CI)									
			Inpatient Services					Outpatient Services				
			Medicine	Nonmedical costs	Diagnostics	Doctors	Other medical costs	Medicine	Nonmedical costs	Diagnostics	Doctors	Other medical costs
Andhra Pradesh	103 114.0	933.7 (0.9)	25.6 (24.9 to 26.3)	21.7 (20.5 to 22.8)	13.6 (13.1 to 14.1)	19.4 (18.6 to 20.2)	19.5 (18.9 to 20.1)	65.4 (63.0 to 67.9)	12.8 (11.0 to 14.7)	10.8 (9.5 to 12.1)	9.9 (9.1 to 10.8)	0.8 (0.4 to 1.2)
Bihar	63 016.2	852.9 (1.4)	33.3 (32.5 to 34.1)	24.6 (23.6 to 25.5)	13.5 (12.9 to 14.0)	12.4 (11.7 to 13.1)	16.0 (15.4 to 16.6)	61.3 (57.5 to 65.2)	13.0 (11.1 to 14.9)	9.2 (7.8 to 10.6)	13.5 (11.8 to 15.2)	2.7 (1.2 to 4.2)
Chhattisgarh	36 945.2	551.7 (1.5)	27.9 (26.5 to 29.2)	43.3 (41.3 to 45.2)	9.0 (8.1 to 9.8)	6.3 (5.6 to 7.0)	13.3 (12.4 to 14.3)	63.9 (59.3 to 68.4)	18.6 (14.5 to 22.6)	3.9 (2.6 to 5.1)	9.9 (8.0 to 11.9)	3.5 (1.8 to 5.2)
Goa	9164.2	126.2 (1.4)	31.7 (27.7 to 35.7)	31.3 (27.0 to 35.6)	7.3 (5.6 to 9.0)	9.8 (6.9 to 12.7)	19.7 (17.1 to 22.3)	46.5 (31.6 to 61.4)	19.2 (−0.7 to 39.1)	15.6 (9.8 to 21.3)	16.9 (10.0 to 23.7)	1.7 (−0.8 to 4.2)
Gujarat	172 649.0	1308.3 (0.8)	25.6 (24.9 to 26.2)	13.4 (12.8 to 14.1)	12.1 (11.7 to 12.4)	25.5 (24.8 to 26.3)	23.2 (22.6 to 23.8)	48.2 (44.7 to 51.6)	9.6 (8.4 to 10.8)	18.6 (16.4 to 20.8)	18.9 (17.0 to 20.8)	4.5 (3.0 to 6.1)
Haryana	84 447.0	519.6 (0.6)	24.6 (23.8 to 25.4)	23.0 (21.6 to 24.3)	12 (11.4 to 12.6)	16.1 (15.2 to 16.9)	24.1 (23.3 to 24.9)	55.3 (52.7 to 57.8)	15.3 (13.2 to 17.3)	12.6 (10.8 to 14.4)	14.4 (12.9 to 16.0)	2.2 (1.5 to 2.9)
Himachal Pradesh	17 985.6	289.1 (1.6)	37.6 (35.7 to 39.6)	25.0 (23.2 to 26.9)	16.6 (15.5 to 17.6)	6.9 (5.7 to 8.1)	13.7 (12.3 to 15.0)	70.7 (63.5 to 77.9)	18.5 (14.0 to 23.0)	3.5 (1.6 to 5.3)	1.1 (0.3 to 1.8)	6.0 (2.7 to 9.3)
Jammu and Kashmir	17 865.5	283.8 (1.6)	39.7 (38.8 to 40.5)	32.9 (32.0 to 33.8)	15.1 (14.5 to 15.6)	1.8 (1.3 to 2.2)	10.3 (9.6 to 11.1)	72.4 (70.3 to 74.5)	19.3 (17.7 to 20.9)	2.1 (1.5 to 2.8)	1.5 (1.0 to 1.9)	4.5 (3.4 to 5.5)
Jharkhand	35 911.6	385.2 (1.1)	34.0 (32.9 to 35.1)	31.0 (29.5 to 32.4)	8.1 (7.6 to 8.5)	11.4 (10.6 to 12.2)	15.4 (14.7 to 16.1)	59.6 (57.0 to 62.2)	16.4 (14.3 to 18.5)	6.6 (5.7 to 7.5)	12.8 (11.4 to 14.2)	4.4 (3.2 to 5.6)
Karnataka	176 485.0	1247.2 (0.7)	28.9 (28.4 to 29.5)	15.3 (14.8 to 15.8)	12.2 (11.9 to 12.5)	19.6 (19.1 to 20.1)	23.6 (23.2 to 24.1)	41.1 (37.9 to 44.4)	16.3 (13.8 to 18.8)	17.2 (15.2 to 19.2)	18.9 (16.5 to 21.4)	6.2 (4.8 to 7.5)
Kerala	91 205.0	1033.6 (1.1)	25.7 (25.1 to 26.3)	17.4 (16.7 to 18.2)	15.1 (14.6 to 15.6)	15.8 (15.2 to 16.5)	25.7 (24.9 to 26.4)	49.4 (46.9 to 52)	19.3 (17.1 to 21.5)	15.0 (13.4 to 16.6)	13.0 (11.8 to 14.1)	3.1 (2.0 to 4.2)
Madhya Pradesh	94 214.8	1032.3 (1.1)	26.0 (25.1 to 26.9)	38.2 (36.8 to 39.6)	9.1 (8.7 to 9.5)	9.4 (8.8 to 9.9)	17.1 (16.4 to 17.7)	46.8 (43.7 to 49.9)	22.7 (19.9 to 25.4)	10.8 (9.4 to 12.2)	11.2 (9.9 to 12.5)	8.3 (6.2 to 10.3)
Maharashtra	313 508.0	2132.8 (0.7)	28.4 (27.9 to 28.9)	18.4 (17.7 to 19.1)	11.5 (11.2 to 11.8)	19.3 (18.8 to 19.8)	22.1 (21.7 to 22.5)	56.0 (54.2 to 57.8)	17.5 (15.9 to 19.1)	9.0 (8.1 to 10.0)	15.1 (14.2 to 16.0)	2.2 (1.6 to 2.7)
Northeastern states	61 969.8	1341.3 (2.1)	33.2 (32.6 to 33.8)	30.6 (30.0 to 31.2)	14.1 (13.7 to 14.5)	7.3 (6.9 to 7.7)	14.6 (14.1 to 15.0)	57.8 (55.3 to 60.3)	19.0 (17.0 to 20.9)	11.4 (9.9 to 12.9)	8.5 (7.3 to 9.7)	3.1 (2.0 to 4.3)
Odisha	56 520.0	690.6 (1.2)	38.0 (36.9 to 39.2)	30.3 (29.1 to 31.5)	11.4 (10.8 to 12.0)	9.2 (8.3 to 10.0)	10.9 (10.2 to 11.5)	64.6 (61.9 to 67.3)	18.7 (16.4 to 21.0)	6.9 (5.8 to 8.0)	6.9 (5.8 to 8.0)	2.6 (1.4 to 3.8)
Punjab	62 222.7	423.5 (0.7)	31.6 (30.7 to 32.6)	16.6 (15.5 to 17.7)	15.1 (14.5 to 15.8)	15.7 (15 to 16.5)	20.7 (19.9 to 21.4)	65.7 (63.3 to 68.1)	9.6 (8.1 to 11.0)	11.0 (9.6 to 12.5)	11.2 (10.2 to 12.2)	2.3 (1.3 to 3.2)
Rajasthan	108 572.0	1352.9 (1.2)	31.2 (30.2 to 32.2)	23.8 (22.5 to 25.1)	15.4 (14.7 to 16.0)	11.0 (10.2 to 11.7)	18.4 (17.6 to 19.2)	48.4 (45.0 to 51.8)	16.5 (13.1 to 19.9)	20.8 (18.2 to 23.4)	12.9 (11.8 to 14.0)	1.1 (0.2 to 2.1)
Tamil Nadu	190 039.0	1580.2 (0.8)	21.6 (20.9 to 22.2)	21.5 (20.5 to 22.4)	12.3 (11.8 to 12.9)	21.4 (20.7 to 22.2)	23 (22.4 to 23.6)	43.7 (41.3 to 46.2)	15.1 (13.6 to 16.6)	17.1 (15.5 to 18.7)	17.3 (16.2 to 18.4)	6.5 (5.0 to 8.1)
Telangana	97 906.5	816.5 (0.8)	23.6 (22.8 to 24.3)	22.4 (21.3 to 23.5)	11.1 (10.7 to 11.6)	24.4 (23.5 to 25.3)	18.2 (17.7 to 18.7)	62.7 (59.7 to 65.6)	10.4 (8.4 to 12.4)	8.3 (7.0 to 9.7)	15.9 (14.5 to 17.3)	2.5 (1.5 to 3.4)
Union territories	29 8611.0	2933.1 (1.0)	23.8 (22.2 to 25.4)	23.1 (20.8 to 25.3)	15.8 (14.4 to 17.2)	15.4 (13.8 to 17.0)	21.7 (20.3 to 23.1)	65.1 (59.1 to 71.1)	12.6 (9.4 to 15.8)	7.8 (5.3 to 10.2)	9.5 (6.3 to 12.6)	4.8 (1.4 to 8.2)
Uttar Pradesh	189 858.0	2310.5 (1.2)	34.1 (33.5 to 34.7)	22.3 (21.5 to 23.0)	11.6 (11.3 to 11.9)	13.5 (13.0 to 14.0)	18.3 (17.8 to 18.7)	66.2 (64.6 to 67.7)	11.4 (10.4 to 12.4)	5.6 (4.9 to 6.2)	12.5 (11.7 to 13.2)	4.1 (3.3 to 5.0)
Uttarakhand	28 968.7	232.4 (0.8)	29.4 (28.2 to 30.7)	27.7 (26.0 to 29.5)	13.2 (12.3 to 14.1)	13.5 (12.3 to 14.8)	15.8 (14.8 to 16.9)	43.2 (37.4 to 48.9)	35.5 (29.1 to 41.9)	5.0 (3.3 to 6.7)	11.0 (8.6 to 13.3)	5.1 (2.9 to 7.4)
West Bengal	129 946.1	1357.5 (1.0)	28.0 (27.0 to 28.9)	31.7 (30.5 to 33.0)	13.2 (12.4 to 13.9)	8.3 (7.6 to 9.0)	18.6 (17.7 to 19.4)	69.8 (67.4 to 72.1)	7.9 (6.7 to 9.0)	9.1 (7.7 to 10.5)	10.8 (9.6 to 12.0)	2.2 (1.3 to 3.1)

^a^ A conversion rate of $1 US = 0.0137 Indian rupees was used.

Mean share of OOPE from medicines was relatively higher among newborns (40.4% [95% CI, 32.4%-48.3%]) in inpatient services, and for skin disease (68.9% [95% CI, 64.5%-73.4%]) in outpatient services. Mean percentages of OOPE from doctor consultation charges were highest for genitourinary diseases (21.3% [95% CI, 20.4%-22.2%]) in inpatient services and blood diseases (20.0% [95% CI, 17.3%-22.7%]) in outpatient services. Share of OOPE from diagnostic tests was 21.6% (95% CI, 15.8%-27.4%) for cancer and 26.2% (95% CI, 21.4%-30.9%) for obstetrics for outpatient services. Nonmedical costs contributions to OOPE from eye diseases were 27.8% (95% CI, 26.1%-29.6%) for inpatient and 17.6% (95% CI, 12.9%-22.3%) for outpatient services (Table 4).

**Table 4.** Proportion of Total Health Expenditure From Service Components by Disease Type

Disease type	Mean proportion, % (95% CI)									
	Inpatient services					Outpatient services				
	Medicine	Nonmedical costs	Diagnostics	Doctors	Other medical costs	Medicine	Nonmedical costs	Diagnostics	Doctors	Other medical costs
Infectious	30.7 (30.3 to 31.0)	19.9 (19.5 to 20.3)	13.1 (12.9 to 13.3)	13.3 (13.1 to 13.5)	22.8 (22.5 to 23.1)	56.9 (55.8 to 58.0)	17.1 (16.2 to 18.0)	8.7 (8.2 to 9.2)	13.4 (12.9 to 13.9)	3.5 (3.1 to 4.0)
Cancer	34.5 (32.6 to 36.4)	18.4 (16.4 to 20.3)	14.5 (13.5 to 15.5)	15.3 (13.7 to 16.8)	17.1 (15.8 to 18.5)	52.7 (45.1 to 60.3)	10.7 (7.9 to 13.4)	21.6 (15.8 to 27.4)	7.9 (6.1 to 9.7)	6.8 (3.4 to 10.3)
Blood diseases	33.3 (31.8 to 34.8)	15.4 (14.1 to 16.8)	12.5 (11.7 to 13.4)	12.6 (11.3 to 13.8)	25.9 (24.5 to 27.3)	44.0 (37.8 to 50.1)	8.9 (6.7 to 11.2)	12.5 (10.3 to 14.6)	20.0 (17.3 to 22.7)	14.3 (10.7 to 18.0)
Endocrine, metabolic, or nutrition	30.2 (29.0 to 31.3)	17.4 (16.0 to 18.7)	14.6 (13.8 to 15.3)	16.4 (15.4 to 17.4)	21.3 (20.4 to 22.2)	60.5 (59.0 to 62.0)	11.2 (10.3 to 12.1)	12.8 (11.9 to 13.6)	12.6 (11.9 to 13.2)	2.7 (2.0 to 3.4)
Psychiatric and neurological	33.9 (33.0 to 34.8)	16.8 (15.9 to 17.7)	15.7 (15.1 to 16.2)	13.7 (13.1 to 14.4)	19.7 (19.0 to 20.3)	64.7 (61.5 to 67.9)	14.0 (11.7 to 16.2)	6.3 (5.0 to 7.6)	10.0 (8.5 to 11.5)	4.8 (3.3 to 6.2)
Genitourinary	28.1 (27.3 to 29.0)	15.1 (14.1 to 16.0)	14.7 (14.0 to 15.4)	21.3 (20.4 to 22.3)	20.5 (19.7 to 21.3)	53.5 (48.1 to 58.9)	14.1 (9.9 to 18.2)	16.1 (13.1 to 19.1)	12.7 (9.8 to 15.7)	3.3 (1.1 to 5.6)
Eye	26.3 (25.0 to 27.7)	27.8 (26.1 to 29.6)	8.3 (7.7 to 9)	17.0 (15.8 to 18.2)	20.2 (19.1 to 21.4)	65.3 (58.6 to 72.0)	17.6 (12.9 to 22.3)	6.0 (3.5 to 8.6)	9.5 (6.7 to 12.3)	1.2 (−0.1 to 2.7)
Ear	34.8 (31.5 to 38.1)	22.2 (18.7 to 25.7)	9.0 (7.6 to 10.4)	16.8 (13.9 to 19.6)	17.0 (14.8 to 19.2)	63.0 (53.1 to 72.9)	14.5 (8.8 to 20.1)	6.2 (2.0 to 10.5)	10.9 (5.6 to 16.2)	5.1 (2.1 to 8.2)
Cardiovascular	29.9 (29.2 to 30.5)	16.6 (15.8 to 17.3)	14.7 (14.3 to 15.1)	16.6 (16.0 to 17.2)	22.0 (21.4 to 22.6)	67.0 (65.5 to 68.5)	12.1 (11.1 to 13.2)	7.9 (7.2 to 8.6)	10.9 (10.2 to 11.7)	1.8 (1.3 to 2.2)
Respiratory	31.6 (30.5 to 32.6)	18.9 (17.7 to 20.0)	13.7 (13.0 to 14.3)	13.7 (12.9 to 14.6)	21.9 (21.0 to 22.8)	67.2 (65.1 to 69.3)	14.2 (12.6 to 15.8)	4.0 (3.3 to 4.6)	11.3 (10.2 to 12.4)	3.2 (2.3 to 4.0)
Gastrointestinal	28.5 (28.0 to 29.1)	20.2 (19.5 to 21.0)	13.8 (13.4 to 14.2)	18.1 (17.5 to 18.7)	19.1 (18.6 to 19.6)	55.3 (52.4 to 58.2)	14.0 (12.0 to 16.0)	15.5 (13.5 to 17.6)	11.8 (10.5 to 13.0)	3.1 (2.0 to 4.2)
Skin	30.1 (28.1 to 32.1)	18.0 (15.9 to 20.2)	14.0 (12.7 to 15.2)	14.6 (13.1 to 16.2)	23.0 (21.2 to 24.9)	68.9 (64.5 to 73.4)	10.1 (7.8 to 12.4)	3.8 (1.9 to 5.7)	13.3 (10.9 to 15.7)	3.6 (1.3 to 5.9)
Musculoskeletal	27.9 (27.0 to 28.8)	17.2 (16.3 to 18.2)	12.8 (12.2 to 13.3)	20.5 (19.5 to 21.4)	21.4 (20.6 to 22.1)	65.3 (63.0 to 67.7)	13.7 (12.2 to 15.2)	7.7 (6.4 to 9.0)	9.8 (8.7 to 11.0)	3.1 (2.3 to 4.0)
Injury	29.5 (28.9 to 30.0)	19.8 (19.1 to 20.4)	11.3 (11.0 to 11.6)	18.0 (17.4 to 18.5)	21.3 (20.8 to 21.7)	57.1 (52.4 to 61.8)	16.6 (12.7 to 20.6)	7.8 (6.0 to 9.5)	12.5 (10.6 to 14.5)	5.7 (3.5 to 8.0)
Childbirth	26.8 (26.5 to 27.2)	32.9 (32.4 to 33.5)	10.5 (10.3 to 10.7)	13.6 (13.3 to 14.0)	15.9 (15.6 to 16.1)	44.1 (11.1 to 77.1)	9.9 (0.4 to 19.3)	12.4 (−0.1 to 25.1)	18.6 (10.1 to 27.1)	14.7 (−1.4 to 31.0)
Obstetric	29.6 (28.6 to 30.6)	21.5 (20.3 to 22.8)	13.4 (12.7 to 14.0)	17.0 (15.9 to 18.0)	18.2 (17.4 to 19.0)	36.1 (30.8 to 41.4)	15.6 (9.3 to 21.9)	26.2 (21.4 to 30.9)	13.9 (10.5 to 17.3)	8.0 (4.7 to 11.2)
Illness in newborn	40.4 (32.4 to 48.3)	16.2 (11.2 to 21.1)	15.3 (11.2 to 19.5)	13.5 (8.3 to 18.7)	14.4 (10.3 to 18.5)	55.3 (45.1 to 65.5)	16.7 (10.7 to 22.6)	11.2 (5.1 to 17.3)	16.2 (9.9 to 22.5)	0.4 (−0.9 to 1.8)
Other	32.0 (30.3 to 33.7)	16.7 (15.2 to 18.1)	14.3 (13.2 to 15.3)	17.3 (15.9 to 18.7)	19.6 (18.4 to 20.7)	51.7 (45.4 to 58.0)	13.0 (8.3 to 17.6)	21.3 (15.7 to 27.0)	12.7 (9.7 to 15.8)	1.0 (0.2 to 1.9)

### Correlation of Health Care Component Costs With State Net Domestic Product per Capita

There was no correlation between share of OOPE from diagnostic testing, doctor consultation charges, other medical costs, or nonmedical costs and state net domestic product per capita for inpatient or outpatient services. Share of OOPE from medicine for outpatient services decreased significantly with increase in state net domestic product per capita (*r* = −0.5 [95% CI, −0.8 to −0.2]; *t* test *P* = .007) (eFigure 2 in the Supplement). Details of social and public health insurance schemes are provided in eTable 6 in the Supplement.

## Discussion

Dissection of OOPE into its components and their relative share may be essential to answer questions such as “What contributes to high OOPE for health care?” and “Where should we focus efforts to reduce OOPE?” Stratification, as done in this cross-sectional study, may provide a preliminary insight into the contributors associated with health expenditure and help guide future research and policy directives. We found that medicines had the largest contributions to OOPE, accounting for up to 60% of OOPE. The share contributed by doctor consultation charges and diagnostic tests ranged from 10% to 15% and were higher for private facilities, urban residents, and individuals aged 65 years and over. Medicines and nonmedical costs contributed a larger share of OOPE in public facilities and among low-income and rural residents. These findings are similar to those in existing literature.^5,6,7,8,9,10,11,12^

The novel findings of this study suggest 4 major patterns. First, medicines continued to be the highest contributor of health care expenditures. States that spent a higher percentage of their GDP on health care had a higher share of OOPE from medicines but a lower share from doctor consultation fees, nonmedical costs, and diagnostic charges. Given that we have undertaken a complete case analysis, a higher proportion of OOPE from medicines does not imply absence of other costs; rather, it may imply cheaper diagnostic tests, doctor consultations, or other health services. Second, nonmedical costs, which are usually ignored by the state, were high. These costs were higher among individuals who were low income, undereducated, members of scheduled tribes, and rural residents and contributed to more than 35% of OOPE in the public sector. Third, diagnostic testing and doctor consultation charges were high for inpatients and in the private sector and increased with socioeconomic status (SES). This may suggest underuse of these services among individuals with low SES or overuse among individuals with high SES. Differential use of public sector services by SES level may be associated with the difference in relative contribution of doctor fees and diagnostic test to OOPE by SES. Additionally, the median analysis highlights that 50% of patients in outpatient settings incurred no costs for diagnostic and doctor consultation charges in public facilities. Fourth, the mean annual expenditure in outpatient settings for medicines, diagnostic testing, and nonmedical services was higher than annual inpatient costs for the same services. However, variations by age, education, income level, and sex were higher for inpatient services.

### Pattern 1: Medicines Dominated Health Expenditure

Although India has one of the lowest levels of cost for medicines in the world^20^ and pharmaceutical underuse is predominant owing to limited or no access to essential medicines for nearly 68% of the population, medicines dominated OOPE.^5,21,22,23^ The availability of free drugs in the pubic setting is low and has declined significantly in the past 2 decades.^24^ Inefficient drug procurement and supply chain management systems are associated with shortages of essential medicines in public health facilities.^22,25,26^ Inelastic health care demand, information asymmetry, skewed power dynamics between buyers and sellers in the drugs market, and the manufacturer-doctor nexus are associated with inhibition of informed choice by the customer and high drug prices.^27,28,29^ Additionally, overprescription and low quality of drugs are associated with irrational use and an accentuated burden of medicine on individuals.^30,31^

The Pradhan Mantri Bhartiya Janaushadhi Pariyojana (2008), recently revamped, aims to provide quality generic medicines at affordable prices. Currently, 8012 Janaushadhi stores provide 1451 drugs and 240 surgical items, which are 50% to 90% cheaper than medicines with the same chemical composition in non-Janaushadhi stores. It has saved up to US $23 million of citizen money on drugs in 2020 to 2021.^32^ However, regulation of the pharmaceutical market is necessary to reduce information asymmetry. To do this and reduce irrational over-the-counter drug use, Janaushadhi stores can collaborate with local doctors to provide information. Websites in local languages along the lines of WebMD that provide information about medicines, their costs, local availability, doctors near the individual, and disease-specific information may foster informed decision-making among patients. In this context, a mobile application and online search tool named Pharma Sahi Daam provides ceiling prices for all scheduled medicines under the National List of Essential Medicines.

### Pattern 2: High Burden of Nonmedical Health Care Costs

Nonmedical costs may be the most underappreciated component of health expenditure. They mainly consist of expenditure on travel, accommodation, and similar items. For half the population, nonmedical services cost up to 37% of OOPE in public facilities and 18% of OOPE for individuals who were low income. They cost a mean of 23.6% for inpatient and 14.6% for outpatient services and 1.7% to 2.7% of annual household income. They were higher among individuals who were less educated, low income, members of scheduled tribes, rural residents, and receiving care in public facilities. Poor health infrastructure and unavailability of diagnostic tests are prime reasons for medical travel.^33,34,35^ This suggests that a dependable primary health care system is necessary for containing nonmedical costs. An efficient primary care system with a higher spectrum of facilities may be associated with effective and early treatment of diseases and improved health outcomes at lower cost.^36^ Furthermore, travel concessions in Indian railways or state-run buses could be offered for medical reasons. Additionally, availability of public-run, cheap lodging services near major hospitals may reduce nonmedical costs.

### Pattern 3: Doctor Consultation Fees and Diagnostic Test Charges and SES

Doctor consultation and diagnostic test charges increased with SES. Nevertheless, their share among individuals with low income was significant too. While doctor fees were minimal in public facilities, their share of OOPE was substantial in a private setting, especially for inpatient services. An increase in doctor fees may be associated with deteriorating doctor-patient relationships and increased doctor shopping, which may be associated with improper diagnosis, ineffective treatments, and increased health care expenditure.^37,38,39^ High doctor consultation charges in private facilities and high rates of private health care use in India^3,37,38^ suggest that the government should work toward a national policy for capping of doctor fees.

Diagnostic testing is another important factor associated with increased OOPE. Availability of diagnostic testing is associated with the quality of care available given that it is essential for correct diagnosis, quality, and efficient care.^33,40,41^ The increasing share of OOPE from diagnostics tests may be associated with wasteful and unnecessary routine tests, screening procedures, and supplier-induced demand of diagnostic tests in India and worldwide.^42,43^ India started the Free Drugs and Diagnostic Service Initiative under the National Health Mission in 2014 to 2015 and recently introduced the essential diagnostic test list. However, considering the decreased availability of free drugs in public facilities,^5,23^ concerns have been raised about the actualization of essential diagnostic test initiatives. For the same reason, the diagnostic and pharmaceutical market may be regulated through national special purpose vehicles.

### Pattern 4: Annual Outpatient Costs Exceeded Annual Inpatient Costs

We compared the relative share of mean annual income expended by mean annual inpatient and outpatient OOPE. Overall, annual inpatient and outpatients costs account for 15.6% and 21.9% of income, respectively. This share was much higher among women and individuals who were low income. Interventions through public-funded health insurance schemes may be effective in reducing OOPE. Given that outpatient services are not covered under health insurance schemes, their burden may be more catastrophic. Currently, few public-funded central or state government health insurance schemes are available in India,^44^ with 10 specifically designed for vulnerable populations. However, except for a few insurance schemes for government employees, no scheme provides reimbursement for outpatient services and travel expenses. The high preference for private facilities in India suggests that insurers should target outpatient costs as well.

### Limitations

This study has several limitations. First, the sample for this study was restricted to individuals who reported expenditures for every component of OOPE. Given that many individuals who reported at least 1 of the costs were excluded from the analysis owing to missing values, the distribution was skewed. Second, all expenditures were self-reported and thus vulnerable to reporting bias, especially for inpatient services, which have a yearlong recall period. Third, a temporal comparison of changes in percentage distribution is absent.

## Conclusions

This study found that medicines contributed the largest share of OOPE. Nonmedical costs were a significant component of health expenditure. Annual outpatient expenditure on medicines, diagnostic tests, and nonmedical costs was higher than annual inpatient expenditure.

## Notes

Supplement. **eTable 1.** Sample Distribution of Individuals by Demographic and Socioeconomic Characteristics **eFigure 1*.*** Sample Selection Flow Chart for Expenditure **eTable 2.** Percent Distribution of Total Health Expenditure Into Components by Traditional Mean Method for Selected Sociodemographic Characteristics **eTable 3.** Components of Health Care Expenditure as a Percent of Total Health Expenditure With ≥1 Missing Value for Household **eTable 4.** Median Percent of Health Expenditure Components for Selected Sociodemographic Characteristics **eTable 5.** Components of Health Care Expenditure as a Percent of Total Income of Household by Selected Sociodemographic Characteristics **eFigure 2.** Association Between Components of Health Care Expenditure and State Net Domestic Product Per Capita at Constant Price 2017-2018 **eTable 6.** Eligibility, Coverage and Benefits of Various National and State Government-Run Health Insurance Schemes
eMethods 1.

eMethods 2.
