# Supplementary material for: Components of Out-of-Pocket Expenditure and Their Relative Contribution to Economic Burden of Diseases in India
Source: JAMA Netw Open. 2022 May 13;5(5):e2210040. doi: 10.1001/jamanetworkopen.2022.10040 (PMC9107026; doi:10.1001/jamanetworkopen.2022.10040)
Supplement: Supplement. — eTable 1. Sample Distribution of Individuals by Demographic and Socioeconomic Characteristics eFigure 1. Sample Selection Flow Chart for Expenditure eTable 2. Percent Distribution of Total Health Expenditure Into Components by Traditional Mean Method for Selected Sociodemographic Characteristics eTable 3. Components of Health Care Expenditure as a Percent of Total Health Expenditure With ≥1 Missing Value for Household eTable 4. Median Percent of Health Expenditure Components for Selected Sociodemographic Characteristics eTable 5. Components of Health Care Expenditure as a Percent of Total Income of Household by Selected Sociodemographic Characteristics eFigure 2. Association Between Components of Health Care Expenditure and State Net Domestic Product Per Capita at Constant Price 2017-2018 eTable 6. Eligibility, Coverage and Benefits of Various National and State Government-Run Health Insurance Schemes eMethods 1. eMethods 2. [file jamanetwopen-e2210040-s001.pdf]

Supplemental Online Content

Ambade M, Sarwal R, Mor N, Kim R, Subramanian SV. Components of out-of-pocket expenditure and their relative contribution to economic burden of diseases in India. *JAMA Netw Open*. 2022;5(5):e2210040. doi:10.1001/jamanetworkopen.2022.10040

eTable 1. Sample Distribution of Individuals by Demographic and Socioeconomic Characteristics

eFigure 1*.* Sample Selection Flow Chart for Expenditure

eTable 2. Percent Distribution of Total Health Expenditure Into Components by Traditional Mean Method for Selected Sociodemographic Characteristics

eTable 3. Components of Health Care Expenditure as a Percent of Total Health Expenditure With ≥1 Missing Value for Household

eTable 4. Median Percent of Health Expenditure Components for Selected Sociodemographic Characteristics

eTable 5. Components of Health Care Expenditure as a Percent of Total Income of Household by Selected Sociodemographic Characteristics

eFigure 2. Association Between Components of Health Care Expenditure and State Net Domestic Product Per Capita at Constant Price 2017-2018

eTable 6. Eligibility, Coverage and Benefits of Various National and State Government-Run Health Insurance Schemes

eMethods 1.

eMethods 2.

This supplemental material has been provided by the authors to give readers additional information about their work.

© 2022 Ambade M et al. *JAMA Network Open.*

eTable 1. Sample Distribution of Individuals by Demographic and Socioeconomic Characteristics[a]

	**Inpatient**		**Outpatient**	
	Sample size	Percentage	Sample size	Percentage
**India**	43,781	100	8,914	100
**Age group**				
0 to 24	12,782	30.7	2,246	29.7
25 to 64	26,830	59.9	4,901	54.2
65 and above	4,169	9.3	1,767	16.0
**Education of patient**				
Below secondary	26,262	64.2	6,406	73.2
Secondary and higher secondary	11,915	25.3	1,693	19.2
Graduate or above	5,604	10.4	815	7.5
**Monthly per capita income (quintiles)**				
Poorest	8,764	24.4	1,847	28.3
Poor	8,919	22.7	1,722	21.2
Middle	9,996	22.3	1,887	18.7
Rich	7,475	15.2	1,676	16.1
Richest	8,627	15.1	1,782	15.4
**Gender**				
Male	16,509	35.6	4,738	51.7
Female	27,272	64.3	4,176	48.2
**Caste**				
Scheduled Tribe	4,473	6.3	626	6.7
Scheduled caste	7,017	18.5	1,449	17.3
Other backward castes	18,437	45.5	3,524	42.5
General	13,854	29.6	3,315	33.2
**Place of residence**				
Rural	24,106	67.0	4,591	63.9
Urban	19,675	32.9	4,323	36.0
**Type of facility**				
Public hospital	16,180	34.8	2,154	20.0
NGO hospital	1103	2.52		
Private hospital	26,498	62.7	2,811	25.9
Private clinic	-	-	3,123	40.7

[a]Percentages given for sociodemographic characteristics are weighted and given with an unweighted sample size, as is typically done because weighted percentages represent population distribution; therefore, calculated weighted percentages may differ from percentages as acquired by dividing numerator by denominator.

© 2022 Ambade M et al. *JAMA Network Open.*

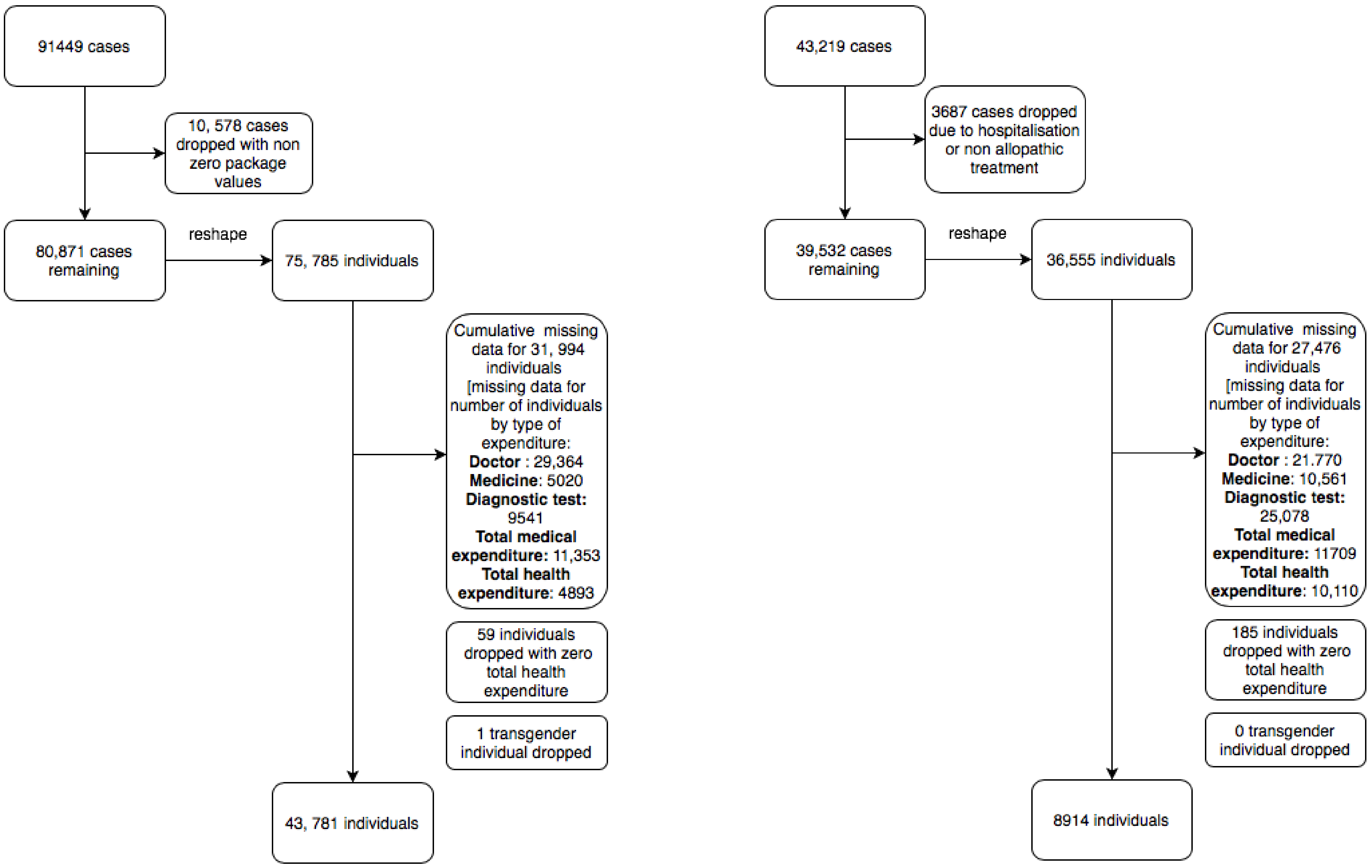

A. Inpatient expenditure

B. Outpatient expenditure

eFigure 1. Sample Selection Flow Chart for Expenditure

© 2022 Ambade M et al. *JAMA Network Open.*

eTable 2. Percent Distribution of Total Health Expenditure Into Components by Traditional Mean Method for Selected Sociodemographic Characteristics

	Inpatient							Outpatient						
	**Sample**	*Total*	*Medicine*	*Nonmedical costs*	*Diagnostic*	*Doctor*	*Other medical*	**Sample**	*Total*	*Medicine*	*Nonmedical cost*	*Diagnostic*	*Doctor*	*Other medical*
**India**	43781	100	28.2	11	12.7	24.2	23.8	8914	100	49.8	12.2	19.1	13.4	5.4
**Age group**														
0 to 24	12782	100	30.1	11.9	12.5	22.2	23.3	2246	100	48.1	12.7	16.7	14.8	7.7
25 to 64	26830	100	27.5	11.1	12.4	24.9	24	4901	100	49.6	11.9	21.4	13	4.2
65 and above	4169	100	28.5	9.3	14	24.2	24	1767	100	52.9	12.8	15.2	12.8	6.3
**Education of patient**														
Below secondary	26262	100	28.9	11.8	12.7	22.8	23.7	6406	100	51.9	13.2	15.5	13.3	6
Secondary and higher secondary	11915	100	28	10.5	12.6	25.3	23.7	1693	100	44.4	10.5	29.5	11.7	3.9
Graduate or above	5604	100	26.4	9.1	12.5	27.4	24.5	815	100	50.5	9.8	14.7	19	6
**Monthly per capita income (quintiles)**														
Poorest														
	8764	100	29.8	13.6	11.8	23.9	20.9	1847	100	52	12.3	21.3	11	3.4
Poor	8919	100	29.2	12.7	12	22	24.1	1722	100	50.5	14.1	16	11.5	7.8
Middle	9996	100	28.5	11.6	12.8	23.7	23.4	1887	100	50.9	14.7	15.8	12.7	6
Rich	7475	100	29	10.6	12.7	23.2	24.5	1676	100	48	8.8	20.4	16.4	6.3
Richest	8627	100	25.6	7.7	13.7	27.4	25.6	1782	100	47.3	11.3	21.4	15.8	4.3
**Gender**														

© 2022 Ambade M et al. *JAMA Network Open.*

Male	16509	100	28.9	10.4	12.6	23.8	24.3	4738	100	48.4	12.2	19.5	13.6	6.4
Female	27272	100	27.5	11.5	12.8	24.7	23.4	4176	100	51.3	12.3	18.7	13.2	4.5
**Caste**														
Scheduled Tribe	4473	100	29.1	16.5	10.1	19.9	24.3	626	100	49.5	16.5	12.4	14.6	6.9
Scheduled caste	7017	100	29.1	12.2	13	22.6	23.1	1449	100	46.8	11.2	25.5	10.6	5.9
Other backward castes	18437	100	28.5	11.1	12.2	24.7	23.5	3524	100	52.2	12.9	16	12.6	6.2
General	13854	100	27.4	9.7	13.4	24.9	24.6	3315	100	48.5	11.3	20.6	15.5	4.1
**Place of residence**														
Rural	24106	100	28.8	26	12.3	23.2	22.9	4591	100	51.9	13.7	17.9	11.4	5.1
Urban	19675	100	27.3	8.4	13.3	25.8	25.2	4323	100	46.9	10.1	20.9	16.1	6
**Type of facility**														
Public hospital/NGO hospital	17283	100	37	26	14.7	5.6	16.4	2,154	100	59	20.5	9.8	3.5	6.9
Private hospital	26498	100	27.3	9.4	12.4	26.2	24.7	2,811	100	45.8	11.6	21.7	14	6.9
Private clinic	-							3,123	100	50.9	10.6	18.7	16.2	3.5

*N***ote:** Values for NGO run hospitals are not provided here for I.P. and NGO and traditional healers for O.P.D service

© 2022 Ambade M et al. *JAMA Network Open.*

eTable 3. Components of Health Care Expenditure as a Percent of Total Health Expenditure With ≥1 Missing Value for Household

	**Inpatient**							**Outpatient**						
	**Sample**	*Total*	*Medicine*	*Nonmedical costs*	*Diagnostic*	*Doctor*	*Other medical*	**Sample**	*Total*	*Medicine*	*Nonmedical cost*	*Diagnostic*	*Doctor*	*Other medical*
**India**	75560	100	28.2(28-28.3)	35.7(35.4-35.9)	10.6(10.5-10.7)	9.9(9.8-10)	15.4(15.3-15.5)	35049	100	63.7(63.4-64.1)	17.6(17.3-17.8)	4.1(3.9-4.2)	10.9(10.7-11)	3.6(3.4-3.7)
**Age group**														
0 to 24	24,572	100	27.3(27.1-27.6)	38.4(37.9-38.8)	9.8(9.7-10)	8.8(8.6-8.9)	15.5(15.2-15.7)	9,617	100	59.9(59.3-60.5)	18.1(17.6-18.7)	4(3.7-4.2)	13.9(13.6-14.3)	3.8(3.5-4.1)
25 to 64	44,335	100	28.4(28.1-28.6)	35.2(34.9-35.5)	10.7(10.6-10.9)	10.3(10.2-10.5)	15.1(15-15.3)	18,288	100	65.4(64.9-65.9)	17.1(16.7-17.5)	4.2(4.1-4.4)	9.7(9.5-10)	3.3(3.1-3.5)
65 and above	6,653	100	30(29.5-30.6)	27.9(27.1-28.6)	12.6(12.3-12.9)	11.9(11.5-12.3)	17.3(16.9-17.7)	7,144	100	65.4(64.6-66.2)	17.8(17.2-18.5)	3.8(3.5-4)	8.8(8.5-9.2)	3.9(3.6-4.3)
**Education of patient**														
Below secondary	19,473	100	28.6(28.4-28.8)	37.9(37.6-38.2)	10.2(10.1-10.3)	8.4(8.3-8.5)	14.7(14.5-14.8)	25,706	100	63.2(62.8-63.6)	18.8(18.4-19.1)	3.7(3.5-3.8)	10.5(10.3-10.7)	3.6(3.4-3.8)
Secondary and higher secondary	19473	100	27.3(27-27.6)	33.2(32.8-33.7)	11.4(11.2-11.5)	11.7(11.5-11.9)	16.2(16-16.4)	6,369	100	64.7(63.9-65.5)	14.9(14.2-15.5)	5.7(5.4-6.1)	11.4(11-11.8)	3.1(2.8-3.4)
Graduate or above	7729	100	26.7(26.3-27.2)	24.2(23.6-24.9)	11.9(11.6-12.1)	17.5(17.1-17.9)	19.4(19.1-19.7)	2,974	100	66.8(65.6-67.9)	11.2(10.5-12)	4.4(4-4.8)	13.4(12.7-14)	4(3.4-4.5)
**Monthly per capita income (quintiles)**														
Poorest	15,122	100	31.1(30.8-31.5)	40.1(39.6-40.6)	9.4(9.2-9.6)	6.7(6.4-6.9)	12.4(12.2-12.7)	7,985	100	64.8(64-65.5)	18.2(17.6-18.8)	3.7(3.5-3.9)	9.1(8.8-9.4)	4(3.6-4.3)
Poor	15,403	100	28.7(28.3-29)	39.5(38.9-40)	9.8(9.6-10)	7.8(7.5-8)	14(13.8-14.3)	7,031	100	61.8(61-62.6)	18.8(18.1-19.5)	3.6(3.3-3.8)	11.4(11-11.7)	4.2(3.8-4.6)
Middle	14,811	100	27(26.6-27.3)	36.4(35.9-36.9)	10.8(10.5-11)	9.8(9.6-10.1)	15.8(15.5-16)	6,018	100	61.7(60.8-62.6)	20.4(19.6-21.1)	4.2(3.9-4.5)	10.5(10.1-10.9)	2.9(2.6-3.3)
Rich	15,115	100	26(25.7-26.4)	33.7(33.1-34.2)	11.2(11-11.4)	11.8(11.6-12.1)	17(16.7-17.3)	7,015	100	63.3(62.5-64.1)	16.3(15.6-16.9)	4.8(4.5-5.1)	11.9(11.5-12.3)	3.4(3.1-3.7)
Richest	15,109	100	26.4(26.1-26.7)	24.2(23.7-24.7)	12.7(12.5-12.9)	16.4(16.2-16.7)	20(19.7-20.2)	7,000	100	66.8(66.1-67.6)	13.1(12.5-13.6)	4.5(4.2-4.8)	12.7(12.3-13.1)	2.6(2.4-2.9)
**Gender**														
Male	26524	100	29.4(29.1-29.7)	29.4(29-29.8)	11.7(11.5-11.8)	11.5(11.4-11.7)	17.7(17.5-17.9)	16661	100	63.1(62.6-63.6)	17.9(17.5-18.3)	4(3.8-4.1)	11.1(10.8-11.3)	3.7(3.5-4)

© 2022 Ambade M et al. *JAMA Network Open.*

Female	49036	100	27.6(27.4-27.8)	38.7(38.4-39)	10.1(9.9-10.2)	9.2(9-9.3)	14.3(14.2-14.5)	18388	100	64.3(63.8-64.8)	17.3(16.9-17.7)	4.2(4-4.3)	10.7(10.4-10.9)	3.4(3.2-3.6)
**Caste**										.				
Scheduled Tribe	9,574	100	25.8(25.3-26.3)	50.7(50-51.4)	7.7(7.5-8)	5.2(5-5.4)	10.3(10-10.6)	2,211	100	53.8(52.3-55.2)	27.2(25.8-28.5)	3.4(3-3.8)	9.9(9.3-10.4)	5.6(4.8-6.3)
Scheduled caste	13,215	100	29(28.6-29.4)	40.2(39.6-40.8)	9.8(9.6-10.1)	7(6.8-7.3)	13.7(13.4-13.9)	5,692	100	64.1(63.2-65)	18.2(17.4-18.9)	4(3.7-4.3)	9.4(9.1-9.8)	4.1(3.7-4.5)
Other backward castes	30,855	100	27.7(27.4-27.9)	35.1(34.7-35.4)	10.6(10.4-10.7)	10.7(10.5-10.8)	15.8(15.6-16)	14,078	100	62.3(61.7-62.8)	19.4(18.9-19.9)	3.9(3.7-4.1)	10.9(10.7-11.2)	3.2(3-3.4)
General	21,916	100	29(28.8-29.3)	29(28.6-29.4)	12(11.8-12.2)	12.2(12-12.5)	17.5(17.3-17.7)	13,068	100	67(66.5-67.6)	13.3(12.9-13.7)	4.4(4.2-4.7)	11.7(11.4-12)	3.3(3.1-3.6)
**Place of residence**														
Rural	44202	100	28.5(28.3-28.8)	38.3(37.9-38.6)	10(9.9-10.2)	8.6(8.4-8.7)	14.3(14.2-14.5)	18067	100	63.6(63.1-64.1)	19.3(18.8-19.7)	3.6(3.5-3.8)	9.7(9.5-9.9)	3.5(3.3-3.7)
Urban	31358	100	27.2(27-27.5)	29.7(29.3-30)	11.8(11.7-12)	13.1(12.9-13.3)	17.9(17.7-18.1)	16982	100	63.9(63.4-64.4)	14.5(14.1-14.9)	4.8(4.6-5)	12.9(12.6-13.2)	3.6(3.4-3.8)
**Type of facility**														
Public hospital	43366	100	27.8(27.6-28.1)	53.5(53.2-53.8)	8.8(8.7-8.9)	0.9(0.8-0.9)	8.7(8.6-8.8)	10030	100	47.6(46.8-48.4)	43.3(42.5-44.1)	3.3(3.1-3.5)	1.4(1.3-1.5)	4.2(3.9-4.5)
Private hospital	30685	100	28.6(28.4-28.7)	12.2(12.1-12.4)	12.9(12.8-13)	21.8(21.6-22)	24.2(24.1-24.4)	9179	100	65(64.5-65.6)	11(10.8-11.3)	6.6(6.3-6.9)	13.8(13.5-14.1)	3.3(3.1-3.6)
Private clinic								12210	100	67.5(67-68)	7.7(7.5-7.9)	4(3.8-4.2)	17.4(17.1-17.7)	3.2(2.9-3.4)

*N***ote:** Values for NGO run hospitals are not provided here for I.P. and NGO and traditional healers for O.P.D services

1

© 2022 Ambade M et al. *JAMA Network Open.*

eTable 4. Median Percent of Health Expenditure Components for Selected Sociodemographic Characteristics

	Inpatient						Outpatient					
	Sample size	*Medicine*	*Nonmedical costs*	*Diagnostic tests*	*Doctor*	*Other medical*	Sample size	*Medicine*	*Nonmedical costs*	*Diagnostic tests*	*Doctor*	*Other medical*
**India**	43,781	26.7(16.1-40)	13.7(7.3-28.3)	10.2(4.1-17.2)	10.7(0-24.4)	18.3(8.1-28.4)	8,914	60.4(40.6-86.9)	7.4(0-18)	0(0-16.1)	9.1(0-21)	0
**Age group**												
0 to 24	12,782	26.7(15.8-39.7)	15.3(8-33.3)	9.4(2.4-16.3)	8.9(0-22.4)	18.1(6.6-28.5)	2,246	58.3(41.1-77.5)	8.7(0-19.3)	0(0-14.8)	13.1(0-23.5)	0
25 to 64	26,830	26.7(16.1-40)	13.4(7.2-27.3)	10.4(4.2-17.4)	11.1(0-25.4)	18.1(8.2-28)	4,901	61.9(40.9-90)	6.8(0-17.1)	0(0-17.2)	8.1(0-20)	0
65 and above	4,169	27(16.4-39.2)	11.5(5.9-21.2)	12(6.3-18.5)	13.2(2-24.7)	20.1(10.9-30.4)	1,767	62.5(38.8-96.1)	6.9(0-19.2)	0(0-13.3)	4.7(0-17.6)	0
**Education of patient**												
Below secondary	26,262	27.7(16.4-41.2)	15.1(8-31.3)	10(3.1-17)	8.8(0-21.5)	17.8(6.2-27.9)	6,406	60.8(41.3-87.5)	8.1(0-19.3)	0(0-14.5)	8.6(0-20.4)	0
Secondary and higher secondary	11,915	25.8(15.6-38.4)	12.9(6.8-26)	10.6(4.9-17.6)	12.5(0-27.7)	18.6(9.3-28.7)	1,693	58.7(38.8-81.4)	6.5(0-15.5)	0(0-20)	10(0-21.7)	0
Graduate or above	5,604	24.4(15.6-35.7)	9.5(5.1-16.8)	10.9(5.9-17.1)	18.4(6.4-33.4)	20.4(12.6-30.3)	815	56.1(34.7-99)	4.6(0-11.8)	0(0-15.1)	14.2(0-25.6)	0
**Monthly per capita income (quintiles)**												
Poorest	8,764	30(16.3-45)	18.7(9.5-41.1)	8.7(0-16.4)	2.9(0-18.6)	13.8(0-24.5)	1,847	62.5(43.4-90.9)	8.3(0-20)	0(0-14.6)	5.7(0-18.1)	0
Poor	8,919	27.7(16.4-40.5)	15.8(8.8-33.9)	9.7(3.2-16.5)	7.5(0-20.6)	17.9(6.5-27.5)	1,722	61.4(42.6-88.2)	9(0-19.3)	0(0-13.7)	8.6(0-18.1)	0
Middle	9,996	26.6(16.4-39.2)	14(7.5-26.3)	10.2(4.2-17.3)	11.4(0-24.2)	19.2(9.6-29.3)	1,887	58.3(38.4-80)	8.6(0-20)	0(0-15)	11.2(0-22.3)	0
Rich	7,475	25.3(15.7-37.1)	11.1(6.3-21)	11.1(5.9-17.6)	15.3(2.7-29)	20(11.2-29.4)	1,676	57.8(38.4-85.7)	5.8(0-14.8)	0(0-20.2)	10.9(0-21.4)	0
Richest	8,627	24(15.5-35)	8.8(4.7-15.6)	12.1(7-19)	18(7.6-32)	22.2(13.7-31.8)	1,782	59.5(36.3-88.2)	5.3(0-13.4)	0(0-18)	13.1(0-25)	0

© 2022 Ambade M et al. *JAMA Network Open.*

**Gender**												
Male	16,509	28(17.7-40.3)	11.7(6.4-21.4)	10.9(5.8-17.6)	12.5(1.9-24.7)	20.2(10.5-30)	4,738	60.7(40.9-88.1)	7.1(0-17.6)	0(0-14.8)	10.1(0-22.2)	0
Female	27,272	26.2(15.1-39.6)	15.3(7.9-33.3)	9.8(2.2-16.9)	9(0-24.2)	17.3(6.2-27.4)	4,176	60(40-85.7)	7.6(0-18.6)	0(0-17.4)	8.3(0-20)	0
**Caste**												
Scheduled Tribe	4,473	26.6(12.8-41.9)	27.7(12.8-54.5)	6.6(0-13.9)	0(0-15.4)	12.6(0-25.1)	626	54.8(29.1-76.2)	13.3(0-31.8)	0(0-11.9)	2(0-18.5)	0
Scheduled caste	7,017	28.3(16.8-42.8)	16.3(8.3-32.9)	9.7(0-17.8)	5.5(0-19.4)	16.8(4.4-27.5)	1,449	60.8(41.1-87.5)	8.5(0-17.6)	0(0-17.9)	5.7(0-17.6)	0
Other backward castes	18,437	26.6(16-39.4)	13.3(7.4-26.6)	10.1(4.5-16.7)	12(0-25.1)	18.6(9.3-28.4)	3,524	60.7(42.8-86.2)	7.2(0-18.9)	0(0-15.2)	10(0-20.8)	0
General	13,854	26.2(16.3-38.6)	11.5(6.2-22.7)	11.3(5.5-18.2)	13.3(0-27.1)	19.3(10.2-29.6)	3,315	60.5(38.4-89.6)	6(0-16.6)	0(0-17.1)	10.9(0-23.2)	0
**Place of residence**												
Rural	24,106	27.6(16.4-40.7)	16.1(9-33.3)	9.8(3-16.8)	8.5(0-22.2)	17.2(6.2-27.1)	4,591	61.5(42.8-88.2)	9(0-20.1)	0(0-14.6)	7.3(0-19.1)	0
Urban	19,675	25.1(15.5-38.2)	9.8(5.1-19.3)	11.1(5.7-18)	14.6(1.8-28.5)	20.5(11.4-30.5)	4,323	58(36-83.3)	4.8(0-14)	0(0-18.5)	12.3(0-25)	0
**Type of facility**												
Public	17,238	29.6(0-50)	37.8(20.3-66.6)	0(0-17)	0(0-0)	2.4(0-18.1)	2,154	62.5(8-90.9)	18.5(3.8-54.6)	0(0-0)	0(0-0)	0
Private hospital	26,543	26(17.7-36.2)	9.7(5.6-15.3)	11.6(7.4-17.3)	18.9(10.2-31.9)	22.3(15.2-30.9)	2,811	53.5(38.4-70.9)	8.4(2.9-16)	9.6(0-21.4)	14.7(6.2-22.8)	0
Private clinic							3,123	60(42.3-80)	5.7(0-14.2)	0(0-18.1)	16.6(3.6-25.6)	0

Note: Values for NGO run hospitals are not provided here for I.P. and NGO and traditional healers for O.P.D services

© 2022 Ambade M et al. *JAMA Network Open.*

eTable 5. Components of Health Care Expenditure as a Percent of Total Income of Household by Selected Sociodemographic Characteristics

					**Inpatient**						**Outpatient**			
	**Sample**	*Total*	*Medicine*	*Nonmedical costs*	*Diagnostic*	*Doctor*	*Other medical*	**Sample**	*Total*	*Medicine*	*Nonmedical costs*	*Diagnostic*	*Doctor*	*Other medical*
**India**	43781	15.6	4.4	1.7	2.0	3.8	3.7	8914	21.9	10.9	2.7	4.2	2.9	1.2
**Age group**														
0 to 24	12782	11.2	3.4	1.3	1.4	2.5	2.6	2246	19.6	9.4	2.5	3.3	2.9	1.5
25 to 64	26830	16.4	4.5	1.8	2.0	4.1	3.9	4901	23.2	11.5	2.7	5	3	1.0
65 and above	4169	23.4	6.7	2.2	3.3	5.7	5.6	1767	21.2	11.2	2.7	3.2	2.7	1.3
**Education of patient**														
Below secondary	26262	15.7	4.5	1.9	2.0	3.6	3.7	6406	21.7	11.3	2.9	3.4	2.9	1.3
Secondary and higher secondary	11915	15.1	4.2	1.6	1.9	3.8	3.6	1693	25.8	11.5	2.7	7.6	3.0	1.0
Graduate or above	5604	16.2	4.3	1.5	2.0	4.5	4.0	815	15.6	7.9	1.5	2.3	3.0	0.9
**Monthly per capita income (quintiles)**														
Poorest	8764	19.7	5.9	2.7	2.3	4.7	4.1	1847	33	17.2	4.1	7.0	3.6	1.1
Poor	8919	16.3	4.8	2.1	2.0	3.6	3.9	1722	25	12.6	3.5	4.0	2.9	1.9
Middle	9996	15.1	4.3	1.7	1.9	3.6	3.5	1887	21.9	11.1	3.2	3.4	2.8	1.3
Rich	7475	15.7	4.6	1.7	2.0	3.6	3.8	1676	21.8	10.5	1.9	4.4	3.6	1.4
Richest	8627	13.5	3.5	1.0	1.8	3.7	3.5	1782	14.3	6.8	1.6	3.1	2.3	0.6
**Gender**														

© 2022 Ambade M et al. *JAMA Network Open.*

Male	16509	21.3	6.2	2.2	2.7	5.1	5.2	4738	19.8	9.6	2.4	3.9	2.7	1.3
Female	27272	12.4	3.4	1.4	1.6	3.1	2.9	4176	24.5	12.6	3.0	4.6	3.2	1.1
**Caste**														
Scheduled Tribe	4473	13.7	4.0	2.3	1.4	2.7	3.3	626	27.1	13.4	4.5	3.4	3.9	1.9
Scheduled caste	7017	16.3	4.7	2.0	2.1	3.7	3.8	1449	25.7	12.0	2.9	6.6	2.7	1.5
Other backward castes	18437	15.9	4.5	1.8	1.9	3.9	3.7	3524	23.6	12.3	3.1	3.8	3.0	1.5
General	13854	15.2	4.2	1.5	2.0	3.8	3.7	3315	18.4	8.9	2.1	3.8	2.8	0.8
**Place of residence**														
Rural	24106	17.2	5	2.2	2.1	4.0	3.9	4591	25.8	13.4	3.5	4.6	2.9	1.3
Urban	19675	13.7	3.8	1.2	1.8	3.5	3.5	4323	18	8.5	1.8	3.8	2.9	1.1
**Type of facility**														
Public hospital	17283	6.1	1.8	1.3	0.7	0.2	0.8	2283	15.7	8.8	3.1	1.4	0.5	1.0
NGO	26498	20	5.4	1.9	2.5	5.2	4.9	2811	33	15.1	3.8	7.2	4.6	2.3
Private clinic	-							3123	19	9.7	2.0	3.6	3.1	0.7

Note: Values for NGO run hospitals are not provided here for I.P. and NGO and traditional healers for O.P.D services

© 2022 Ambade M et al. *JAMA Network Open.*

eFigure 2. Association Between Components of Health Care Expenditure and State Net Domestic Product Per Capita at Constant Price 2017-2018

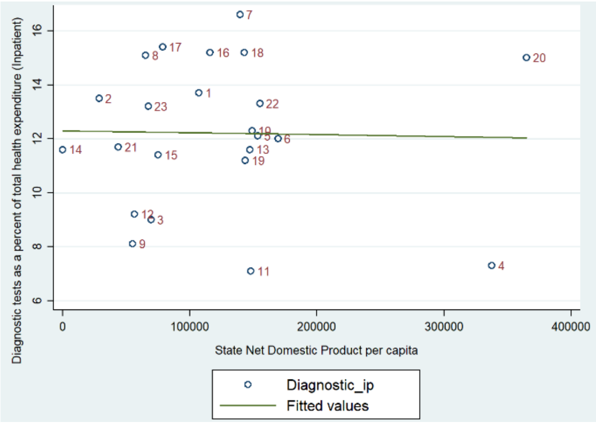

Fig e2 (a): Diagnostic test fee (IP)
[Spearman's correlation coefficient=-0.02; p= 0.92

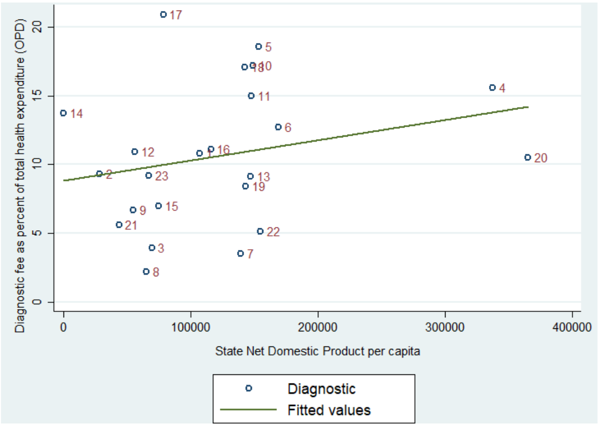

Fig e2 (b): Diagnostic test fee (OPD)
[Spearman's correlation coefficient = 0.25; p= 0.2]

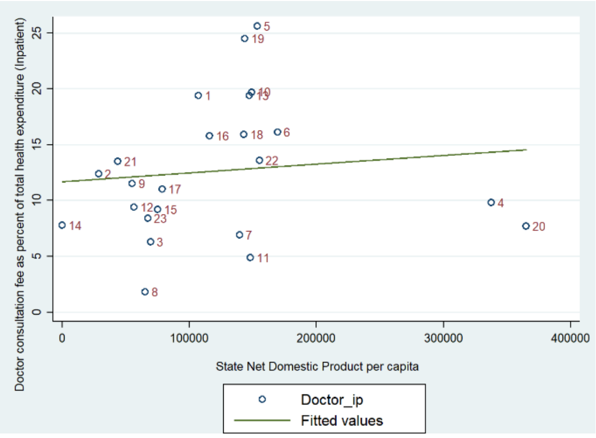

Fig e2 (c): Doctor consultation fee (IP)
[Spearman's correlation coefficient = 0.1; p= 0.6]

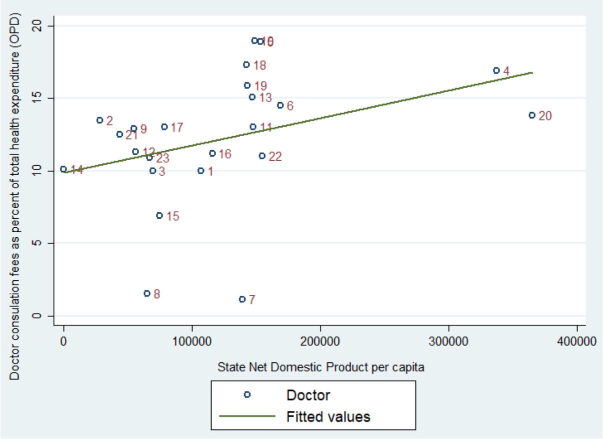

Fig e2 (d): Doctor consultation fee (OPD)
[Spearman's correlation coefficient = 0.35; p= 0.09]

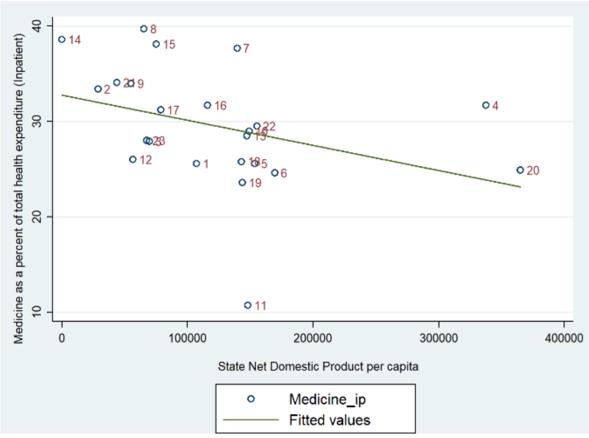

Fig e2 (e): Cost of medicines (IP)
[Spearman's correlation coefficient = --0.35; p= 0.09]

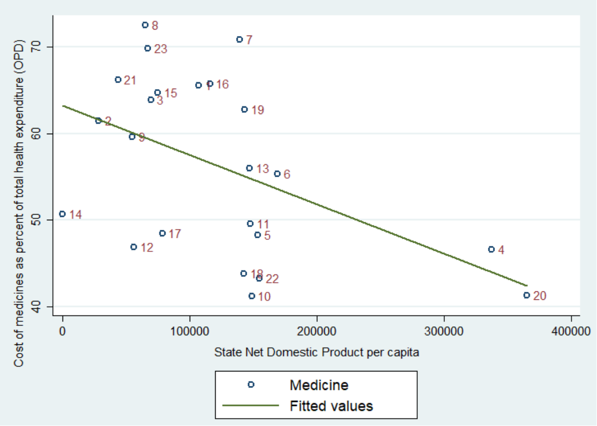

Fig e2 (f): Cost of medicines (OPD)
[Spearman's correlation coefficient = -0.48; p<0.05]

© 2022 Ambade M et al. *JAMA Network Open.*

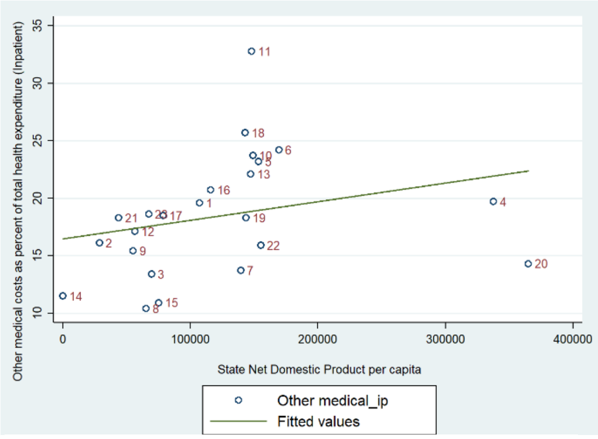

Fig e2 (g): Other medical costs (IP)
[Spearman's correlation coefficient = 0.26; p= 0.2]

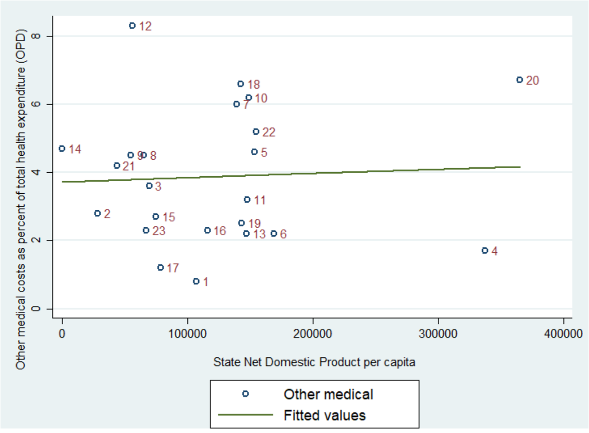

Fig e2 (h): Other medical costs (OPD)
[Spearman's correlation coefficient = 0.05; p= 0.8]

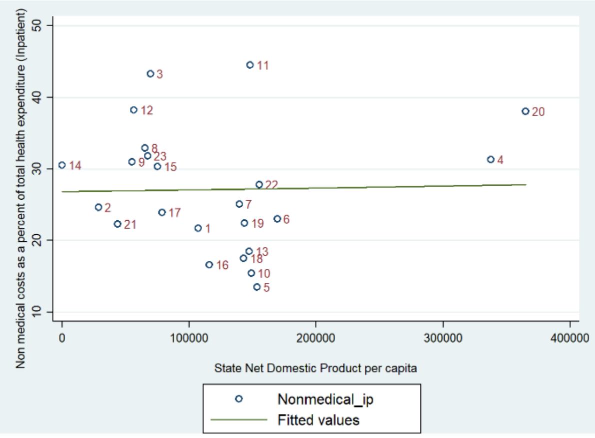

Fig e2 (i): Non-medical costs (IP)
[Spearman's correlation coefficient = 0.02; p= 0.9]

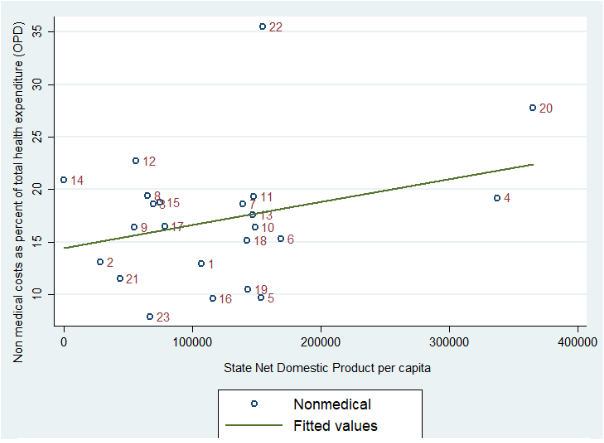

Fig e2 (j): Non-medical costs (OPD)
[Spearman's correlation coefficient = 0.30; p= 0.15]

Note: States have been provided individual ids in the scatterplot.

State	ID	State	ID
Andhra Pradesh	1	Maharashtra	13
Bihar	2	North eastern states	14
Chhattisgarh	3	Odisha	15
Goa	4	Punjab	16
Gujarat	5	Rajasthan	17
Haryana	6	Tamil Nadu	18
Himachal Pradesh	7	Telangana	19
Jammu and Kashmir	8	UTs	20
Jharkhand	9	Uttar Pradesh	21
Karnataka	10	Uttarakhand	22
Kerala	11	West Bengal	23
Madhya Pradesh	12		

© 2022 Ambade M et al. *JAMA Network Open.*

eTable 6. Eligibility, Coverage and Benefits of Various National and State Government-Run Health Insurance Schemes

Name of health insurance scheme	Year	Eligibility	Coverage	Benefits	Other Details
**Central Government Insurance**					
Pradhan Mantri Jan Arogya Yojana	2018	Poor (based on Socio-Economic Caste Census, 2011) and families covered under RSBY	INR 500,000 per family per year	1393 procedures covering all costs related to treatment (drugs, supplies, diagnostics, doctor fee, room charges, OT and ICU charges). All pre-existing conditions covered from day one. Covers up to 3 days of pre hospitalization and 15 days post hospitalization expenses such as diagnostics and medicines	For secondary and tertiary care; Subsumed Rashtriya Swasthya Bima Yojana
Central Government Health Scheme	1954	Central government employees and pensioners	All health expenses	OPD treatment including medicines, cashless facility available for treatment in empanelled hospitals and diagnostics centres, travel expenses reimbursed, reimbursement of expenses of treatment at government /private hospitals under emergency, medical appliances purchase reimbursement	38.5 lakh beneficiaries covered in 74 cities all over India
Employee State Insurance Scheme	1948	All government employees, in government establishment with at least 10 employees and drawing a salary up to INR 15,000	All health expenses	Sickness, disablement, maternity benefits, dependent benefits, funeral expenses	

© 2022 Ambade M et al. *JAMA Network Open.*

Pradhan Mantri Surakhsa Bima Yojana	2015	Aged 18 to 70 years, Indian residents with bank accounts	INR 200,000 for accidental death and full disability and INR 100,000 for full disability	Accidents	GST is exempted, amount is automatically debited from account. Has one-year coverage from June 1 to May 31 and administered through public sector general insurance companies
Universal Health Insurance Scheme	2003	Families below poverty line	INR 30000 for hospitalization floated among entire family along with death cover of INR 25,000	Accidents	
State Government Schemes					
Tamil Nadu: Chief Ministers Comprehensive Insurance Scheme	2012	Resident of Tamil Nadu through name in family card, with a certified annual income less than INR 72,000. Migrants from other states can join with a request letter provided they have six months of residency in the state	INR 500,000 per family per year on floater basis for limited ailments and procedures	All hospitalization coverage	Launched through United India Insurance Company
Rajasthan: Bhamashah Swasthya Bima Yojana	2015	Resident of Rajasthan	Up to INR 300,000 coverage with seven-day pre hospitalisation and 15 day post hospitalisation coverage. Transport allowance of up to INR 500 is provided	Cashless services to IPD patients with access to cashless facilities for 1045 packages for general illness and 500 for critical illness. The packages include bed charges, consultation fee, boarding charges, consumables, diagnostic test and ambulatory services	Insured through New India Assurance Company
Kerala: Karunya Health Scheme	2012	Resident of Kerala with Aadhaar card	Coverage up to INR 300,000 covered	Critical illness such as cancer, haemophilia, kidney	Available at all government and some empanelled private hospitals

© 2022 Ambade M et al. *JAMA Network Open.*

				diseases, heart diseases and palliative care are covered	
Kerala: Awaz health Insurance Scheme	2017	5 lakh inter state labourers living in Kerala (aged 18 to 60)	Health insurance cover up to INR 15,000 and accidental death cover up to INR 200,000	Hospitalisation costs in all government and certain private empanelled hospitals	Migrant workers need to enrol and get a health cared
Maharashtra: Mahastma Jyotiba Phule Jan Aarogya Yojaja	2012	Families holding Yellow ration card, Antyodaya Anna Yojana ration card, Annapurna Ration card, orange ration card with annual income less than INR 100,000, journalists and their dependent family members approved by DGIPE, construction workers and their families have live registration with Maharashtra Building and other construction worker welfare board	Coverage of INR 250,000 per family per year	End to end cashless services for certain diseases in limited government and private empanelled hospitals	
Gujarat: Mukhyamantri Amrutam Yojana	2012	Low- and middle-income families	INR 500,000 per family per annum, INR 399 for travel per hospitalisation with repatriation of INR 6 per kilometre from hospital to the place of residence, all benefit packages of PM-JAY	Cardiovascular surgeries, neurosurgery, burns, ploy trauma, cancer, renal diseases, neonatal diseases are covered, general surgery, knee and hip replacement, mental disorders, obstetric and gynaecology. All hospitalisation costs covered.	
Telangana State Government Employees and Journalists Health Scheme	2017	Employees and pensioners of government of Telangana except those covered under other schemes such as		Hospitalisation cover	

© 2022 Ambade M et al. *JAMA Network Open.*

		Central government health scheme, ESIS etc			
Karnataka: Yeshasvini Health Insurance Scheme	2013	Farmers in the state and informal workers in the lower middle income and middle-income groups, up to age 75 years	Covers 803 surgical procedures. Does not include diagnostic investigation, burns cases, chemotherapy, implants, autoimmune diseases, follow up treatment, cosmetic surgery, dialysis, dental surgeries, kidney and heart transplant	572 network hospitals provide the services	
West Bengal Health for All Employees and Pensioners Cashless Medical Treatment Scheme	2014	State government employees and their families, All India services officers and pensioners	Cashless indoor treatment up to INR 100,111, reimbursement for IOPD treatment and expenses for empanelled hospitals outside West Bengal.	1000 medical procedures	
Andhra Pradesh: Dr. YSR Aarogyasri health care	2007	All BPL families identified by BPL ration card issues by Civil Supplies Department	Coverage up to INR 500,000 for each family per year		Implemented through Dr.YSR Aarogyasri Health Care Trust

© 2022 Ambade M et al. *JAMA Network Open.*

eMethods 1.

National Sample Survey 2017-18 (round 75): Health Consumption

The 75th round of National Sample Survey on health consumption is a nationwide sample survey conducted by Government of India between July 20177 to June 2018. This survey covered 113,823 sample households and 555,115 individuals (Rural :325, 883, Urban: 229,232; Male: 283200, Female: 271,877) from randomly selected 8077 villages and 6181 urban wards using a two-stage random sampling method. The survey was conducted through in-depth interviews of selected individuals at their home.

This survey oversamples those who were hospitalised in the past 365 days and 60+ population. It collects information related to demographic details, household characteristics, morbidity and mortality, hospitalization in the last 365 days, health insurance coverage, out-of-pocket expenditure (OOPE), healthcare utilization, immunization coverage, maternal health, and elderly health. For outpatient visits, survey considered 15 days recall period in self-reporting of ailments. One of the reasons for choosing 15 days recall period over monthly recall (30 days) was to reduce the recall bias in reporting the ailments. All those who are unable to provide bifurcated costs, provide a single total amount categorised as "Package component"

All inpatient and outpatient costs are bifurcated into the a few categories: medicine, doctor consultation charges, diagnostic test charges, hospital bed charges (only for inpatients), other medical charges, non-medical charges (further categorised as travel and lodging and "others").

For inpatient, annual costs are provided for each case of hospitalisation. For outpatients' costs incurred in the past 15 days are provided for each case of doctor visit in the past 15 days. Monthly total household expenditure (which is considered as a proxy for income of the household) is provided for use. Since the study uses annual costs, we have extrapolated the monthly income to annual income.

Study sample and participant selection

Initially, inpatient and outpatient costs were available for 91,449 and 43,219 cases of hospitalization and ailment, respectively. However, many cases had missing values for the doctor, medicine, diagnostics and other medical costs as they reported one single number under the package component. These cases accounted overall for 10% of the inpatient sample. As we aim to bifurcate the costs, cases reporting costs under the package component were dropped from the analysis.

Next, we reshaped the data to provide costs per person, as the unit of analysis was a case of ailment for hospitalization. After reshaping, we replaced missing values for all cases, excluding the first one, with zero. This allowed us to add up costs across cases for each individual. Next, we replaced the missing values for the first cases with zero if that particular case had received the given service free of charge. Then we added up the costs across all cases for each individual. Finally, those individuals with at least one missing value for any of the costs, namely, doctor, medicine, diagnostic, other medical and non-medical, were dropped from the analysis. Thus, the final sample had information about 43,781 individuals hospitalized in the past 365 days and 8914 individuals who reported ailment in the past 15 days (before the survey)

Missing values and selection bias

As we restricted our sample to those who reported costs for all components of health care, we missed information from those who reported costs for some of the components. A significant portion of missing values was noted under doctor costs, especially among public services users. As doctor fees are primarily covered under registration costs in public health care centres, higher missing cases for doctor fees were expected. Due to the varied number of missing cases across costs, we faced a major

© 2022 Ambade M et al. *JAMA Network Open.*

dilemma while selecting the study participants. All the missing values generated for those who did not incur a cost as they did not avail a certain service such as diagnostic test or "other medical costs" could either be replaced with zero or dropped entirely from the analysis. The thought process for the first option revolved around considering that every patient is a customer and health services are goods that the customer may or may not choose to purchase. Under this line of thought, we would replace all missing values due to non-use of service with zero. However, in reality, patients seldom choose a health service. Such decisions are primarily made by the person they are consulting. Therefore, we decided to drop all those cases instead. This decision was taken despite losing information of those who availed at least one service and reported their costs. However, this decision is justified as the inclusion of these individuals would have inflated the relative contribution of those components, which have low non-missing values. Figure 1 presents the sample selection flow diagram.

The components of health care expenditure

The primary outcome of this study is the cost of various components of health care and its relative contribution to the total cost. The total health expenditure was divided into five segments: doctor, medicine, diagnostics, other medical and non-medical expenses. Other medical costs include but are not limited to attendant charges, physiotherapy, personal medical appliances, blood, and oxygen. While information about hospital bed charges was collected for inpatients separately, we clubbed it together with other medical costs to keep the categories identical for inpatient and outpatient services. Non-medical costs include registration fee, food, transport for others, expenditure on patient's travel, lodging charges, if any, etc. Medicine expenses were bifurcated into AYUSH and on AYUSH for outpatient care. Since we dropped non-allopathic care users from outpatient analysis, we did not consider AYUSH medicine cost in this analysis. Information about each inpatient cost was collected for all hospitalization cases per person in 365 days. Similarly, for outpatient costs, data was collected for all spells of ailments per person in the past 15 days. Therefore, we added the costs for all episodes and cases to get prices per individual. We also replaced all missing values with zero if the given service was acquired but free of charge. All the remaining observations with any missing value were dropped from the analysis.

eMethods 2.

The ethnic groups classified as "General", "Other backward castes", "Scheduled Castes (SC)" and "Scheduled tribes (ST)" are classification of Indian population as provided in the constitution of India. The constitution has categorised certain sections of population as "scheduled castes" who were historically underprivileged and faced severe socio-economic discrimination and hardships. The Indian subcontinent followed a form of class division called "casteism" that divided the population into four broad groups. The lowest caste was called the "untouchables". Since these groups were geographically, culturally and etymologically diverse, they were identified and clubbed into a single category-SC. Similarly, the tribal population of India which are geographically and culturally diverse but have a common nature of residing in forests, have been clubbed together under the category "Scheduled tribes". Besides, the SC/ST population, there were other population that was socio-economically marginalised but not "untouchables" or "tribes". They were categorised as "Other backward castes". The remaining population is clubbed under the category "General"

© 2022 Ambade M et al. *JAMA Network Open.*
